# Daily Patterns in the Timing of Medication and Supplement Intake

**DOI:** 10.3390/nu18193291

**Published:** 2026-10-07

**Authors:** Tyler Tran, Shweta Varshney, Laura van Rosmalen, Jordan L. Levy, Kyla L. Laing, Emily N. C. Manoogian, Michael T. Y. Lam, Satchidananda Panda

**Affiliations:** 1 Regulatory Biology Laboratory, The Salk Institute for Biological Studies, 10010 North Torrey Pines Road, La Jolla, CA 92037, USA; tytran@salk.edu (T.T.); svarshney@salk.edu (S.V.); lrosmalen@salk.edu (L.v.R.); jlevy@salk.edu (J.L.L.); emanoogian@salk.edu (E.N.C.M.); 2 Division of Pulmonary, Critical Care, Sleep Medicine, and Physiology, University of California, 9500 Gilman Drive, La Jolla, CA 92093, USA; mtlam@health.ucsd.edu

**Keywords:** chronotherapy, circadian rhythm, supplements, medications, chronopharmacology

## Abstract

**Background/Objectives:** The timing of medication intake can influence therapeutic efficacy and adverse effects, yet little is known about when medications are actually taken in daily life. We aimed to characterize real-world temporal patterns of medication and dietary supplement use at the population level. **Methods:** We analyzed time-stamped medication and dietary supplement records from 26,069 adults enrolled in the myCircadianClock smartphone application, generating a population-scale atlas of real-world intake timing. **Results:** Across more than 200,000 intake events, medication use exhibited daily patterns, with most therapeutic classes following one of two dominant patterns: morning- or evening-predominant intake. In contrast, dietary supplements displayed four distinct temporal patterns, largely corresponding to morning, meal-associated, or bedtime use. Medication timing was highly consistent across age and sex, although the types of medications consumed varied substantially with age. While the timing of many medications aligned with general chronotherapy recommendations, every therapeutic class exhibited considerable variability, with a substantial proportion of intake events occurring several hours outside the predominant intake window. **Conclusions:** This population-scale atlas provides the first comprehensive description of real-world medication and dietary supplement timing. These findings suggest that temporal adherence may be considered an important dimension of medication-taking behavior.

## 1. Introduction

Nearly every aspect of human physiology, including cardiovascular function, endocrine signaling, immune activity, metabolism, gastrointestinal function, renal clearance, and behavior, exhibits robust 24 h circadian rhythms orchestrated by the central and peripheral molecular clocks [1,2,3]. These rhythms regulate the expression of a large part of the genome, including those encoding drug targets, transporters, metabolizing enzymes, and signaling pathways, thereby creating predictable daily variation in both pharmacokinetics and pharmacodynamics. Consequently, administering the same drug dose at different times of day can result in markedly different therapeutic efficacy, toxicity, and overall patient outcomes [4,5,6].

Over the past five decades, evidence supporting chronotherapy has accumulated across multiple therapeutic areas. Experimental studies have demonstrated circadian variation in drug absorption, distribution, metabolism, and elimination [4,5,6], while clinical trials have shown clinically meaningful time-of-day effects across diverse therapeutic areas. Short-acting statins lower LDL cholesterol and triglycerides more effectively when taken in the evening [7], glucocorticoids are most effective when aligned with endogenous inflammatory rhythms [8,9,10], antihypertensive medications often provide greater cardiovascular benefit when administered at bedtime [11,12,13], and appropriately timed chemotherapy can improve efficacy while reducing toxicity [14,15]. A recent review of more than one hundred clinical trials concluded that approximately three-quarters reported significant time-of-day differences in treatment outcomes, underscoring the broad clinical relevance of circadian medicine [16].

Despite these advances, circadian medicine has not been widely implemented in routine clinical practice. Only a minority of commonly prescribed medications include explicit time-of-day recommendations in regulatory labeling, and medication timing is seldom incorporated into clinical guidelines. Much of the existing literature has focused on predicting the optimal time for drug administration under controlled experimental conditions [17,18]. However, drug responses in daily life are influenced not only by endogenous circadian rhythms but also by environmental and behavioral factors, including light exposure, sleep-wake behavior, physical activity, meal timing, and work schedules, which are rarely captured in laboratory studies. Moreover, patients often take medications according to convenience, daily routines, work and sleep schedules, meal timing, symptom severity, caregiver availability, all of which may differ substantially from recommended dosing times. Understanding the temporal organization of medication use under real-world conditions is therefore essential for translating chronotherapy into clinical practice.

Population-scale data on medication timing conveys how medications are used, particularly as the prevalence of chronic disease and polypharmacy continues to rise [19,20]. More than half of adults regularly take one or more prescription medications, and many older adults consume multiple medications and dietary supplements each day [21,22]. These medications are commonly prescribed for chronic conditions that themselves exhibit strong circadian variation—including hypertension, asthma, diabetes, arthritis, gastroesophageal reflux disease, depression, and cardiovascular disease [23,24,25,26]. Yet the temporal organization of medication use remains largely uncharacterized.

The widespread adoption of smartphone-based health applications now enables the collection of time-stamped medication records at unprecedented scale under real-world conditions [27,28]. Unlike prescription databases or pharmacy dispensing records, digital self-report platforms capture the actual time medications are consumed, providing an opportunity to characterize medication timing behavior across populations. Such data can reveal temporal patterns shared across therapeutic classes, identify medications with highly stereotyped or highly variable dosing schedules, quantify demographic differences in medication timing, and establish the extent to which real-world behaviors align with existing chronotherapy recommendations.

Here, we leverage real-world datasets of time-stamped medication intake collected through the myCircadianClock smartphone application to construct an atlas of medication timing in free-living adults. We characterize the daily temporal distribution of dozens of prescription medications and dietary supplements, identify dominant dosing patterns across therapeutic classes, quantify variability in medication timing, and examine differences according to age and sex. Rather than asking *when medications should be taken*, our study addresses the previously unexplored question of when medications are actually taken. By establishing the temporal landscape of real-world medication use, this work provides an empirical foundation for future evaluation of adherence to chronotherapy recommendations, identifying opportunities for circadian optimization of pharmacotherapy, and advancing precision medicine by integrating not only what medications patients take, but also when they take them.

## 2. Methods

### 2.1. Study Overview

This exploratory observational study investigated the circadian timing of medication and dietary supplement intake using data collected through the myCircadianClock (mCC) smartphone application (Android versions 1.0.0–16.9.94; iOS versions 1.0.0–7.5.3). The study was approved by the Salk Institute Institutional Review Board (15-0003) and was renewed annually. The mCC app is a non-commercial research smartphone application designed primarily to collect the timing of dietary intake over several days or weeks [29]. It is available free of charge for Android and iPhone smartphones and has been adapted for numerous observational and lifestyle intervention studies [30,31,32,33,34,35,36,37,38,39]. For the present study, the application was configured for web-based or in-app consent to support large-scale observational data collection. During onboarding, participants entered demographic information including age, and sex. Participants were instructed to maintain their usual lifestyle and record all lifestyle events over a two-week baseline period. Automated reminders encouraged compliance but did not provide behavioral coaching. Participants were unable to review previously entered records during the initial observation period, minimizing behavioral changes resulting from self-monitoring. Only data from this initial two-week period were analyzed because subsequent entries may have been influenced by personalized feedback provided by the application.

### 2.2. Participants

Participants were recruited between 9 September 2015 and 19 February 2024 through the myCircadianClock.org website, smartphone application, public lectures, flyers, social media, and community outreach. Eligible participants were adults (≥18 years) able to provide informed consent and record lifestyle data in English. There were no geographic restrictions. Following electronic informed consent, each participant received a unique activation code for the mCC application. Baseline demographic characteristics are summarized in Table S1. Participants were instructed not to intentionally modify their eating patterns, medication schedules, supplement use, sleep, or physical activity during the baseline recording period so that the collected data reflected habitual behavior. Participants were periodically reminded to log their food and beverage intakes.

### 2.3. Lifestyle and Medication Data Collection

Participants recorded lifestyle information continuously for fourteen consecutive days [40]. The principal objective of the application was comprehensive capture of dietary intake; however, participants could additionally record water intake, medications, dietary supplements, sleep, exercise, and health metrics. Intake events could be logged by photographing the item with an accompanying text description, entering a text description alone, or retrospectively entering the actual time of consumption if immediate recording was not feasible. If internet connectivity was unavailable, entries remained securely stored on the device until transmission was possible. All data were encrypted during transmission. Each record retained both the participant-recorded ingestion time and the server receipt time. Analyses used the participant-recorded local ingestion time because it most accurately reflected biological exposure. Participants were instructed to assign each entry a log type according to its primary purpose. Medication and supplement events were recorded using the medication (‘m’) log type, allowing these records to be distinguished from food entries that contained ingredients resembling supplement names. Once submitted, entries could not be modified or deleted, preserving an immutable audit trail.

### 2.4. Data Quality Control

Participants logging at least one energy-containing food or beverage log and at least one medication or supplement record in the mCC app within the first 14 days were included in the analyses. Consistent with previous temporal analyses of data collected through our app [30,31,32,33,34,35,36,37,38,39,40,41,42] a day extended from 04:00:00 to 03:59:59 the following calendar day to avoid splitting nocturnal eating episodes across calendar days. Delayed logs were defined as records in which the difference between participant-recorded ingestion time and server receipt time exceeded fifteen minutes [40]. A total of 15.31% of logs are delayed logs. Minute-level distributions of delayed logs were examined to distinguish uploads delayed because of internet connectivity from retrospective backlog entries reconstructed from memory. Preferential clustering at rounded five-minute intervals identified probable backlog entries. Chi-square comparisons between delayed and real-time logging distributions were used to objectively identify retrospective entries, with a statistically significant difference between the two (*χ*^2^ = 3431.57, *df* = 59, *p* < 0.001). Therefore, we considered the 37.78% of delayed logs occurring at minutes with large differences in logging preference as backlogs, accounting for 5.80% of total data. Sensitivity analyses comparing cohorts before and after exclusion of users with exclusively delayed logs demonstrated negligible effects on overall timing distributions, supporting robustness of the quality-control strategy.

### 2.5. Medication Extraction and Pharmacologic Classification

Medication identification was performed using an automated natural-language-processing pipeline built around Gemini 2.5 Flash. Unique free-text medication logs were processed once to identify medication entities, correct spelling, normalize abbreviations, and convert proprietary brand names to their corresponding generic active ingredients. Occasional contextual information including dosage, formulation, administration route, treatment indication, and temporal descriptors was removed because these features were not required for timing analyses and they were sparse. Generic disease descriptions or nonspecific medication class descriptions were excluded. A total of 99.98% of unique participant logs were successfully parsed by Gemini, 43.61% of which contained at least one identified medication. Validation of medication parsing was performed using a random sample of 400 participant logs across five independent test runs. Human annotation identified medications in 209 logs and no identifiable medications in 191 logs. Across the five runs, Gemini achieved a mean total accuracy of 96.76%, correctly identifying logs with medications with a mean recall of 95.79% and logs without medications with a mean accuracy of 98.01%. Mean precision was 98.14%, with a mean F1 score of 96.95%. The normalized medication phrases were subsequently mapped to RxNorm _20AA_260406F accessed on 15 April 2026 using the approximate Term interface, which performs spelling correction, stemming, abbreviation expansion, and flexible word-order matching. Candidate concepts were re-ranked using the native RxNorm similarity score together with semantic embedding similarity and lexical similarity. The highest-confidence concept was retained. Established Pharmacologic Class (EPC) classification for these concepts was found by decomposing the concept into active ingredients (other RxNorm concepts), which were mapped to EPC terminology through RxNorm. Terms that could not be confidently normalized or without a mappable EPC were excluded from downstream analyses. The complete language-model prompts are provided in the Supplementary Materials.

### 2.6. Supplement Extraction

Dietary supplements were extracted independently using a second Gemini 2.5 Flash workflow optimized for supplement terminology. The pipeline identified vitamins, minerals, probiotics, fish oil, botanical products, amino acids, and other nutritional supplements while excluding caloric foods such as protein shakes. Marketing language, wellness claims, dosage units, and frequency descriptors were removed. Prescription and over-the-counter medications were ignored unless they also existed as recognized dietary supplements. Validation of supplement parsing was performed using a separate random sample of 400 participant logs across five independent test runs. Human annotation identified supplements in 257 logs and no identifiable supplements in 143 logs. Across the five runs, Gemini achieved a mean total accuracy of 92.70%, correctly identifying logs with supplements at a mean recall of 88.79%. Logs without supplements had a mean accuracy of 99.72%. Mean precision was 99.83%, with a mean F1 score of 93.98% across test runs. Because no universally accepted ontology analogous to RxNorm exists for dietary supplements, supplement ingredients were analyzed individually without additional pharmacologic classification.

### 2.7. Definitions and Statistical Analysis

The primary timing metric was TF50, defined as the median clock time at which 50% of all recorded intake events occurred for an individual medication, EPC, or dietary supplement across the complete observation period. The modality of each temporal distribution was evaluated using Hartigan’s dip test, which tests whether intake times were consistent with a single peak or showed evidence of multiple distinct timing patterns. Continuous variables were summarized using means ± standard deviations or medians with interquartile ranges, as appropriate, whereas categorical variables were summarized as counts and percentages. Temporal distributions were visualized using circular plots, density distributions, and heat maps where appropriate. Hierarchical clustering was performed on 24 h intake timing distributions generated by binning EPC or supplement-specific intake events into 30 min intervals. Similarity among temporal distributions was assessed using the resulting 48-bin profiles, and the number of clusters was selected based on the largest linkage-distance separation observed in the hierarchical clustering dendrogram and the number of clusters was selected based on the largest linkage-distance separation that avoided excessive fragmentation into small clusters. Between-group comparisons employed parametric or non-parametric statistical tests according to data distribution. All statistical tests were two-sided, and *p* < 0.05 was considered statistically significant. Data processing, language-model inference, RxNorm normalization, and statistical analyses were performed using reproducible computational pipelines. Complete computational prompts and implementation details are provided in the Supplementary Materials to facilitate reproducibility.

## 3. Results

### 3.1. Participant Characteristics and Medication/Supplement Record

Applying the inclusion criterion of at least one energy-containing food or beverage and one medication or dietary supplement log during the first 14 days of app use yielded a cohort of 26,069 adults (8276 men and 17,743 women; participant characteristics are summarized in Table S1). Collectively, these participants recorded 2,138,687 mCC logs (mean = 82.04, sd = 67.19 logs/user). Of these, 218,879 (mean = 8.40, sd = 11.39 logs/user) corresponded to medication or supplement logs, which formed the basis for all subsequent analyses. Most participants reported relatively few unique medications or supplements. More than half (52.43%) of participants logged a single medication and nearly three-quarters reported no more than two medications, whereas supplement use was somewhat more diverse, with 38.49% of participants taking one supplement and a larger proportion reporting multiple supplements (Figure S1). The most frequently logged medications were grouped into Established Pharmacologic Classes (EPCs) or analyzed individually (Tables S2 and S3).

### 3.2. Real-World Medication Intake Exhibits Daily Rhythms and Distinct Temporal Dosing Patterns

**Figure 1:**
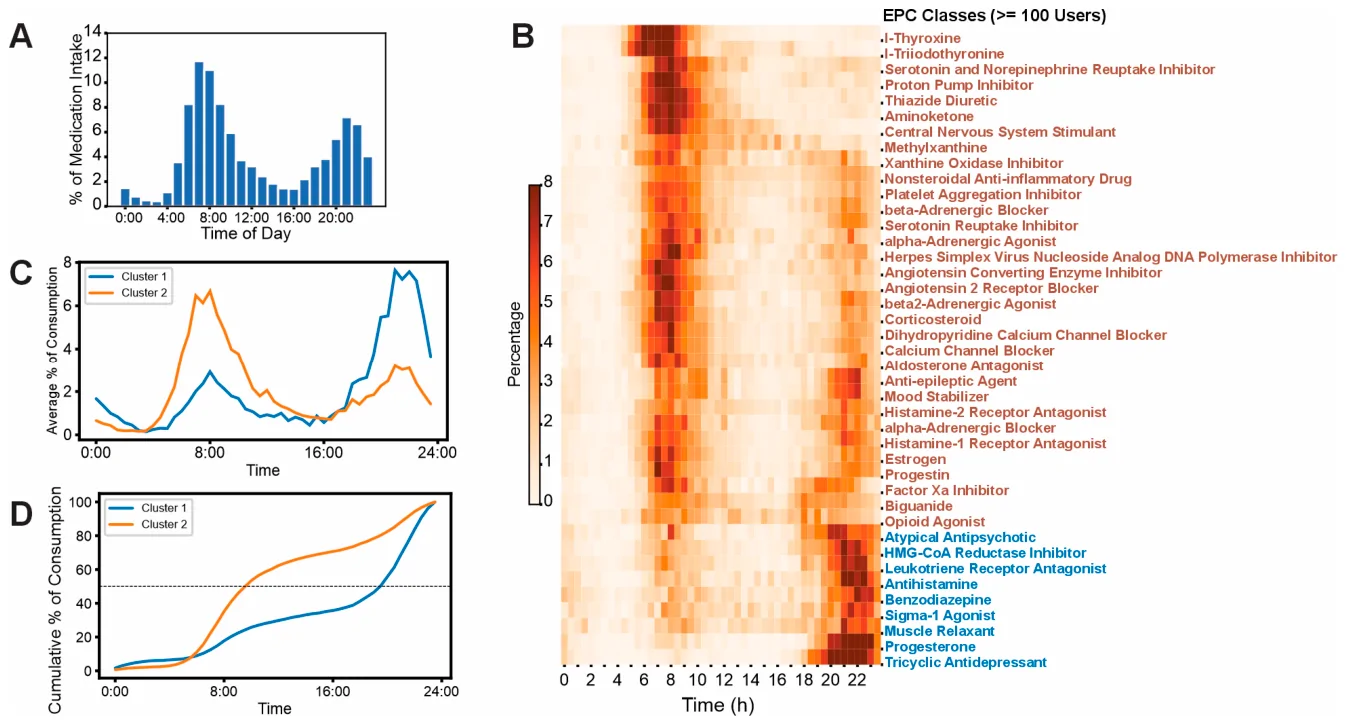
Population-scale temporal patterns of medication intake. (**A**) The distribution of all medication intake events across the 24 h day in 1 h bin. (**B**) A heatmap showing the temporal distribution of intake for the most frequently used Established Pharmacologic Class (EPC) medication classes (≥100 users). Each row represents one EPC and each column represents 30 min of the day. Color intensity denotes the percentage of daily intake occurring within each 30 min interval. Medication classes are ordered by similarity in temporal intake profiles. Labels are color-coded according to the dominant temporal cluster identified by hierarchical clustering. (**C**) The mean 30 min intake profiles for the two major temporal clusters identified from the EPCs. Cluster 1 (orange) exhibits morning dosing preference with a modest evening peak, whereas Cluster 2 (blue) is characterized by predominant evening dosing with a smaller morning peak. (**D**) The corresponding cumulative distribution of medication intake for the two temporal clusters.

**Figure 2:**
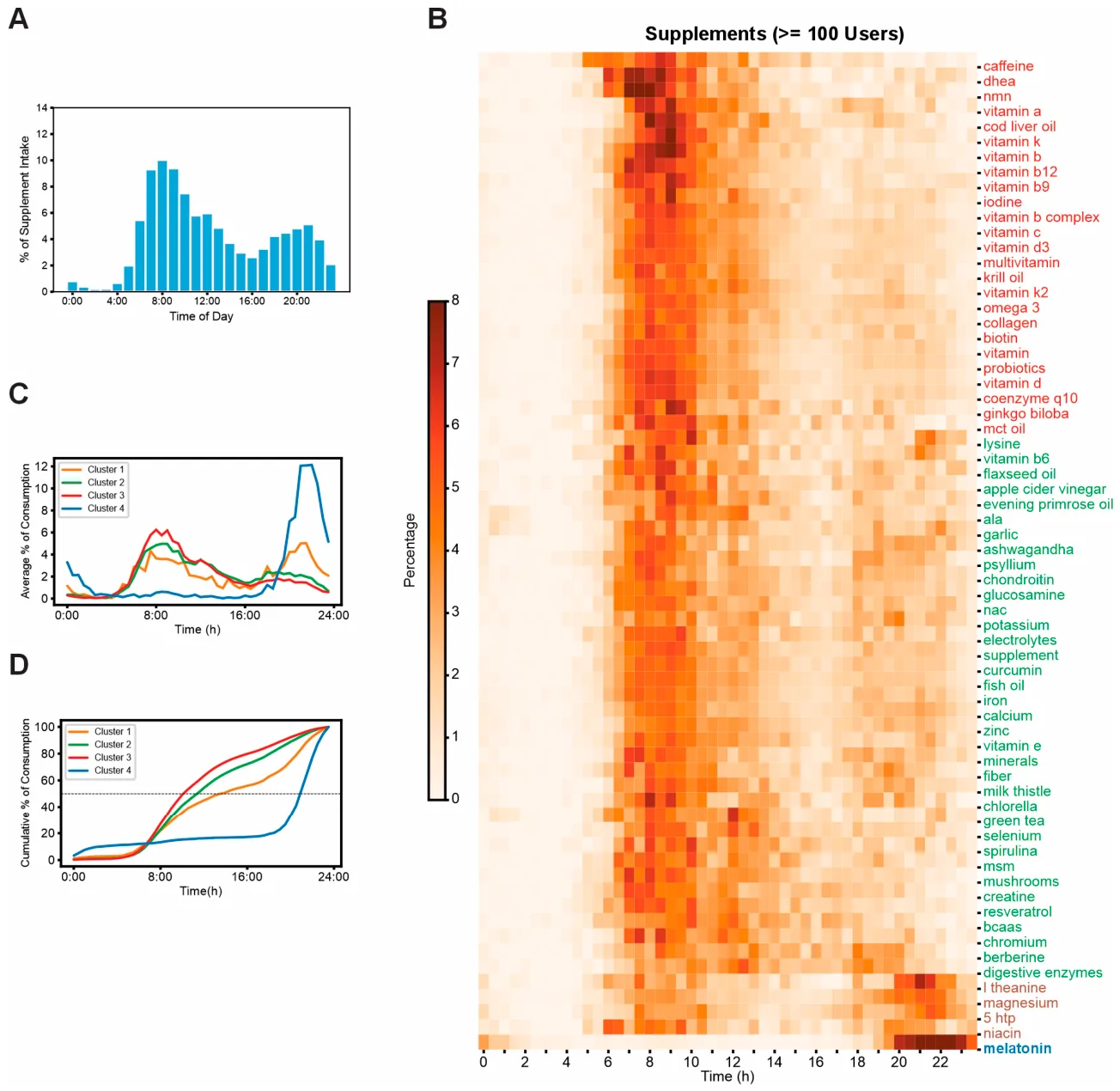
Population-scale temporal patterns of dietary supplement intake. (**A**) The distribution of all dietary supplement intake events across the 24 h day in 30 min bin. (**B**) A heatmap showing the temporal distribution of intake for the most frequently reported dietary supplements (≥100 users). Each row represents one supplement and each column represents 30 min of the day. Color intensity indicates the percentage of daily intake occurring within each 30 min interval. Supplements are ordered by similarity in temporal intake profiles using hierarchical clustering. Label colors denote the four major temporal clusters identified by clustering analysis. (**C**) The mean 30 min intake profiles of the four supplement clusters. Cluster 1 (orange) consists of supplements with a predominant morning peak and modest evening use. Cluster 2 (green) includes supplements exhibiting a morning peak followed by a gradual decline throughout the day. Cluster 3 (red) comprises supplements with a sharp morning peak and sustained daytime intake. Cluster 4 (blue) contains supplements with a dominant evening or bedtime dosing pattern and minimal daytime use. (**D**) The corresponding cumulative intake distributions for the four temporal clusters. Clusters 1–3 accumulate 50% of daily intake during the morning hours. Cluster 4, consisting of only melatonin, shows minimal daytime accumulation and a rapid increase during the late evening.

Because previous studies have shown that many individuals routinely remain awake beyond midnight [43] and that overall human activity reaches its daily nadir between 02:00 and 04:00 [44], we defined each 24 h day as extending from 04:00 to 03:59:59 the following day. Similar to previously reported temporal patterns of food intake [35,40,41,42], medication and supplement intake exhibited daily rhythms (Figure 1A and Figure 2A). Logging frequency was lowest overnight for both medications and supplements, with fewer than 2% of either record type occurring in any hourly bin between 00:00 and 05:00. Intake increased rapidly after approximately 05:00, reaching a major peak between 07:00 and 09:00, which accounted for 22.77% of all medication records and 19.34% of all supplement records. Following this peak, logging gradually declined throughout the afternoon before increasing again during the evening, producing a smaller secondary peak between approximately 20:00 and 22:00 (Figure 1A). When the day was divided into four six-hour intervals, medication intake was strongly concentrated during the morning, with 44.05% of records occurring between 04:00 and 10:00. In comparison, 18.66% of medication records were logged between 10:00 and 16:00, 23.38% between 16:00 and 22:00, and only 13.90% between 22:00 and 04:00.

To determine whether individual therapeutic classes followed common temporal patterns, we examined the 41 most frequently used Established Pharmacologic Classes (EPCs), each consumed by at least 100 users (Figure 1B). Despite the diversity of therapeutic indications, medication classes clustered into two principal temporal profiles (Figure 1C,D). The first cluster with 32 EPCs was characterized by a pronounced morning dosing pattern, with most intake occurring shortly after awakening and cumulative intake reaching 50% by 09:57 and 75% by 20:51. Temporal distributions in this cluster had a median Hartigan’s dip statistic of 0.046 (IQR, 0.029–0.062). The dip statistic quantifies the maximum difference between the observed distribution and the closest-fitting unimodal distribution; values closer to zero indicate greater consistency with unimodality, whereas larger values indicate stronger departures from a single-mode distribution. Because the magnitude of the statistic depends partly on sample size, statistical evidence against unimodality was assessed using the corresponding *p* values. Overall, 28 of the 32 EPCs showed statistically significant evidence of departure from unimodality. This cluster included thyroid hormones, proton pump inhibitors, thiazide diuretics, serotonin and norepinephrine reuptake inhibitors, aminoketones, central nervous system stimulants, corticosteroids, and several antihypertensive drug classes (Figure 1B). In contrast, the second cluster with 9 EPCs exhibited a modest morning peak, reaching 50% by 20:29, followed by a dominant evening peak, resulting in a substantially delayed cumulative intake profile. The median dip statistic across the nine EPCs in this cluster was 0.033 (IQR, 0.031–0.046), with 7 EPCs showing statistically significant evidence of departure from unimodality. (Figure 1C,D). Medications within this cluster included statins, benzodiazepines, muscle relaxants, tricyclic antidepressants, progesterone, antihistamines, leukotriene receptor antagonists, sigma-1 agonists, and atypical antipsychotics (Figure 1B). These findings demonstrate that, at the population level, real-world medication timing is organized into a limited number of circadian dosing behaviors.

Examination of individual EPCs further highlighted the diversity of dosing schedules underlying these two clusters (Figure S2). Several medication classes exhibited highly synchronized morning intake. L-thyroxine showed the most concentrated timing, with 25%, 50%, and 75% of daily consumption occurring within a narrow interval between approximately 06:30 and 9:15. Although Hartigan’s dip test detected a statistically significant departure from unimodality, the small dip statistic indicated that this deviation was modest (dip = 0.008, *p* < 0.001) consistent with a dominant morning peak accompanied by relatively sparse intake events later in the day, and with recommendations for fasting administration before breakfast. Similarly, thiazide diuretics, proton pump inhibitors, central nervous system stimulants, aminoketones, and serotonin-norepinephrine reuptake inhibitors displayed steep cumulative curves and predominantly morning dosing with median intake between 09:00 and 09:52, and 75% of intake occurring by 11:50–14:28. Dip statistics ranged from 0.007 to 0.059, reflecting variation from strongly concentrated morning distributions to more complex profiles with secondary intake periods. Other classes demonstrated broader distributions across the waking day. Biguanides, beta blockers, calcium channel blockers, platelet aggregation inhibitors, and nonsteroidal anti-inflammatory drugs exhibited both morning and evening peaks with median intake times between 10:29 and 14:17 and 75th-percentile intake occurring between 19:29 and 20:15. Their larger dip statistics (0.034–0.055; all *p* < 0.001) were consistent with more gradual cumulative intake curves consistent with twice-daily or flexible dosing schedules. Within the second cluster, progestins, estrogens, and histamine-1 receptor antagonists also exhibited marked morning-evening bimodality: with TF50 occurring between 10:58 and 11:57, and 75th-percentile intake between 20:54 and 21:11, with dip statistics of 0.068–0.082 (all *p* < 0.001). In contrast, HMG-CoA reductase inhibitors were preferentially consumed during the evening, with cumulative intake remaining relatively flat during the daytime before increasing to reach TF50 at approximately 20:00 and 75% of intake by 21:59 (dip = 0.046, *p* < 0.001).

Analysis of the 20 most frequently reported individual medications demonstrated similar patterns (Figure S3). Drugs with established recommendations for morning administration—including levothyroxine, hydrochlorothiazide, omeprazole, fluoxetine, and amphetamine/dextroamphetamine—showed highly concentrated morning dosing with little evening use. Their median intake times ranged from 07:38 to 09:48, with 75% of doses occurring by 09:15 for levothyroxine and by 11:31–13:31 for the other medications. Levothyroxine exhibited the narrowest morning distribution, with the 25th, 50th, and 75th-percentiles of intake occurring at 06:33, 07:38, and 09:15, respectively. Accordingly, though its dip test indicated a statistically significant departure from unimodality, the small dip statistic suggested that this deviation was modest (dip = 0.008, *p* <0.001). Omeprazole and fluoxetine also showed evidence of non-unimodal intake distributions, with dip statistics of 0.016 and 0.029, respectively (both *p* < 0.01), whereas hydrochlorothiazide and amphetamine/dextroamphetamine had small, nonsignificant dip statistics (dip = 0.008 and 0.009; *p* = 0.863 and 0.656), consistent with predominantly unimodal morning dosing. In contrast, medications such as metformin, amlodipine, metoprolol, sertraline, cetirizine, and ibuprofen displayed broad bimodal distributions reflecting substantial use during both morning and evening hours. Median intake occurred between 10:27 and 14:20, while 75th-percentile intakes occurred between 19:25 and 20:33. These medications generally had larger and statistically significant dip statistics, ranging from 0.024 for ibuprofen to 0.061 for cetirizine (all *p* < 0.001), supporting more complex or bimodal intake patterns. Finally, rosuvastatin, montelukast, atorvastatin, and especially progesterone exhibited dominant evening or bedtime administration. Rosuvastatin and montelukast reached median intake at approximately 20:00, with 75% of doses occurring by 21:50 and 22:06, respectively, while atorvastatin had a somewhat earlier median intake time of 18:59 and a 75th percentile of 21:39. Their dip statistics ranged from 0.045 to 0.056 (all *p* < 0.001), consistent with distinct evening peaks alongside smaller daytime intake periods. Progesterone demonstrated the greatest temporal synchronization among the medications examined: the 25th, 50th, and 75th percentiles occurred at 19:59, 21:27, and 22:31, respectively, indicating that the central 50% of doses were taken within an approximately 2.5 h evening window. Its smaller but statistically significant dip statistic (dip = 0.031, *p* < 0.001) suggests a strongly concentrated evening distribution with only limited secondary timing structure. Collectively, these analyses indicate that while overall medication intake follows a common population-level daily rhythm, individual medications and therapeutic classes exhibit distinct temporal signatures that likely reflect a combination of pharmacological recommendations, disease-specific symptom timing, and habitual patient behavior.

### 3.3. Medication Timing Relative to Individual Sleep-Wake Schedules

Because clock time does not account for interindividual differences in sleep-wake schedules, we next expressed medication intake relative to each participant’s self-reported wake time and bedtime (Table S4). Aligning medication timing to wake time revealed even stronger synchronization for several medications. For example, levothyroxine was taken an average of 1.14 h (SD, 1.72 h) after waking, with a median interval of only 0.53 h, highlighting its highly stereotyped administration soon after awakening. Hydrochlorothiazide, omeprazole, and bupropion were typically consumed an average of within 2.42–2.66 h of wake time, consistent with morning dosing recommendations. In contrast, medications such as metformin and ibuprofen were taken substantially later, averaging approximately 4.86 h after waking, with median intervals of 3.50–4.10 h, reflecting their administration with meals or according to symptoms rather than immediately after awakening. Thus, normalizing medication timing to wake time reduces interindividual variability and better captures medications that are tightly linked to the beginning of the biological day.

A complementary analysis aligned medication intake relative to bedtime to identify drugs preferentially administered before sleep. Consistent with the clock-time analyses (Figure 1 and Figure S3), only a subset of medications exhibited synchronization to bedtime (Table S5). Progesterone and rosuvastatin were among the medications taken closest to bedtime, with median intervals of 0.79 and 1.40 h. Together, these analyses demonstrate that medication timing is organized around daily behavioral anchors—awakening and bedtime. Morning medications are generally synchronized to the onset of the active phase, whereas bedtime medications exhibit considerably tighter alignment to sleep onset, suggesting that behavioral routines are a major determinant of real-world medication timing.

### 3.4. Dietary Supplements Exhibit More Diverse Temporal Intake Patterns than Medications

In contrast to medications, dietary supplement intake exhibited a morning preference and greater diversity in temporal intake patterns (Figure 2A). Supplement intake exhibited a broader temporal distribution, with 36.86% of records between 04:00 and 10:00, 30.83% between 10:00 and 16:00, 24.60% between 16:00 and 22:00, and just 7.73% between 22:00 and 04:00. These findings demonstrate that medication and supplement intake follow robust daily patterns characterized by a pronounced morning dosing preference, minimal overnight use, and a smaller evening peak.

Hierarchical clustering of the most frequently consumed supplements (≥100 users) (Table S6) identified four distinct temporal intake patterns (Figure 2B–D). Two large clusters were characterized primarily by morning intake, but differed in the breadth of their intake distributions. Cluster 1, comprising 25 supplements such as multivitamins, vitamins D, D3, B complex, B12, vitamin C, and cod liver oil, exhibited the earliest and most concentrated temporal profile, reaching 50% of daily intake by 10:59 and 75% of intake by 15:42. (Figure 2B). Cluster 2 (36 supplements) consisted largely of nutritional and musculoskeletal supplements, including fish oil, calcium, zinc, iron, glucosamine, chondroitin, curcumin, fiber, selenium, creatine, and digestive enzymes, which also peaked during the morning, reaching 50% intake by 12:03 but showed a broader distribution extending into the afternoon with 75% intake by 18:00 (Figure 2B,C). The remaining clustered exhibited stronger evening preferences. Cluster 3 contained four supplements, magnesium, L-theanine, niacin, and 5-hydroxytryptophan, and displayed a mixed daytime-evening pattern. It reached median intake at 16:48 and 75% intake by 20:56. In contrast, melatonin stood out as its own cluster, showing a strongly synchronized late-evening profile with a median intake at 21:47 and 75th-percentile intake occurring by 22:54. These differences are most apparent in the cumulative intake of these clusters: with two reaching half their total intake by late-morning or mid-day, whereas later clusters are characterized by more gradual accumulation and stronger rises in the evening onward.

Inspection of individual supplement profiles demonstrated considerable variation in the degree of temporal synchronization (Figure S4). Vitamin D, vitamin D3, vitamin B12, multivitamins, probiotics, and coenzyme Q10 exhibited highly synchronized morning intake, with median intake times ranging from 10:09 to 11:10 and 75% of intake occurring between 13:49 and 16:46. Their dip statistics were generally small, ranging from 0.0054 to 0.0151, consistent with strongly concentrated morning distributions. Evidence of departure from unimodality was statistically significant for vitamin D, vitamin D3, multivitamins, probiotics, and coenzyme Q10 (all *p* ≤ 0.001), but not for vitamin B12 (dip = 0.0054, *p* = 0.278), indicating that the latter was particularly consistent with a single dominant morning peak. Vitamin B complex exhibited a similarly narrow morning profile, with 25%, 50%, and 75% of intake occurring at 08:33, 10:56, and 14:41, respectively, and no significant evidence of departure from unimodality (dip = 0.0058, *p* = 0.506). In contrast, fish oil, collagen, iron, zinc, curcumin, and general vitamin supplements displayed broader cumulative curves, indicating intake distributed across breakfast, lunch, and dinner. Fish oil, iron, zinc, and curcumin had median intake times between 11:42 and 12:37, with their 75th-percentile intake occurring between 17:30 and 18:42. Each showed statistically significant evidence of departure from unimodality, with dip statistics ranging from 0.0130 to 0.0198 (all *p* < 0.001), consistent with intake distributed across multiple periods of the day. Collagen also showed a broad cumulative distribution, with median intake at 11:35 and 75% by 16:50, although its small, nonsignificant dip statistic suggested no strong evidence of distinct secondary peaks (dip = 0.0076, *p* = 0.487). Magnesium exhibited pronounced morning-evening bimodality, with the 25th, 50th, and 75th percentiles occurring at 09:40, 17:00, and 20:56, respectively. It also had the largest dip statistic among the evaluated supplements (dip = 0.0452, *p* < 0.001), supporting the presence of distinct morning and evening intake periods. Melatonin, by comparison, was almost exclusively consumed in the late evening, with 25%, 50%, and 75% of intake occurring at 20:39, 21:47, and 22:54. Its small and nonsignificant dip statistic (dip = 0.0079, *p* = 0.140) was consistent with a single, highly synchronized bedtime peak rather than a multimodal distribution. (Figure S4).

To account for interindividual variation in sleep schedules, supplement timing was also expressed relative to awakening and bedtime (Table S7). Most commonly consumed supplements were taken 6–7 h before bedtime, consistent with administration during the morning or midday. For example, multivitamins and vitamin D were consumed, on average, approximately 6.55 h (SD, 3.81; median 7.07 h) and 6.40 h (SD 3.92 h; median 7.05 h) before bedtime, whereas melatonin was taken only an average of 1.08 h (SD 1.25 h; median 0.77 h) hour before sleep, confirming its tight coupling to bedtime behavior. Relative to awakening, supplements generally exhibited greater variability than medications, reflecting their frequent consumption with meals rather than immediately after waking. An exception was magnesium, which was consumed, on average, approximately 4.53 h (SD, 3.68 h; median 3.37 h) after awakening, substantially later than many vitamins and consistent with its mixed daytime and evening use. Together, these findings demonstrate that dietary supplements are organized into multiple conserved temporal behaviors, with timing influenced not only by circadian routines but also by intended physiological function and meal-associated dosing habits.

### 3.5. Age Has a Greater Influence than Sex on Medication Timing and Medication Use Patterns

**Figure 3:**
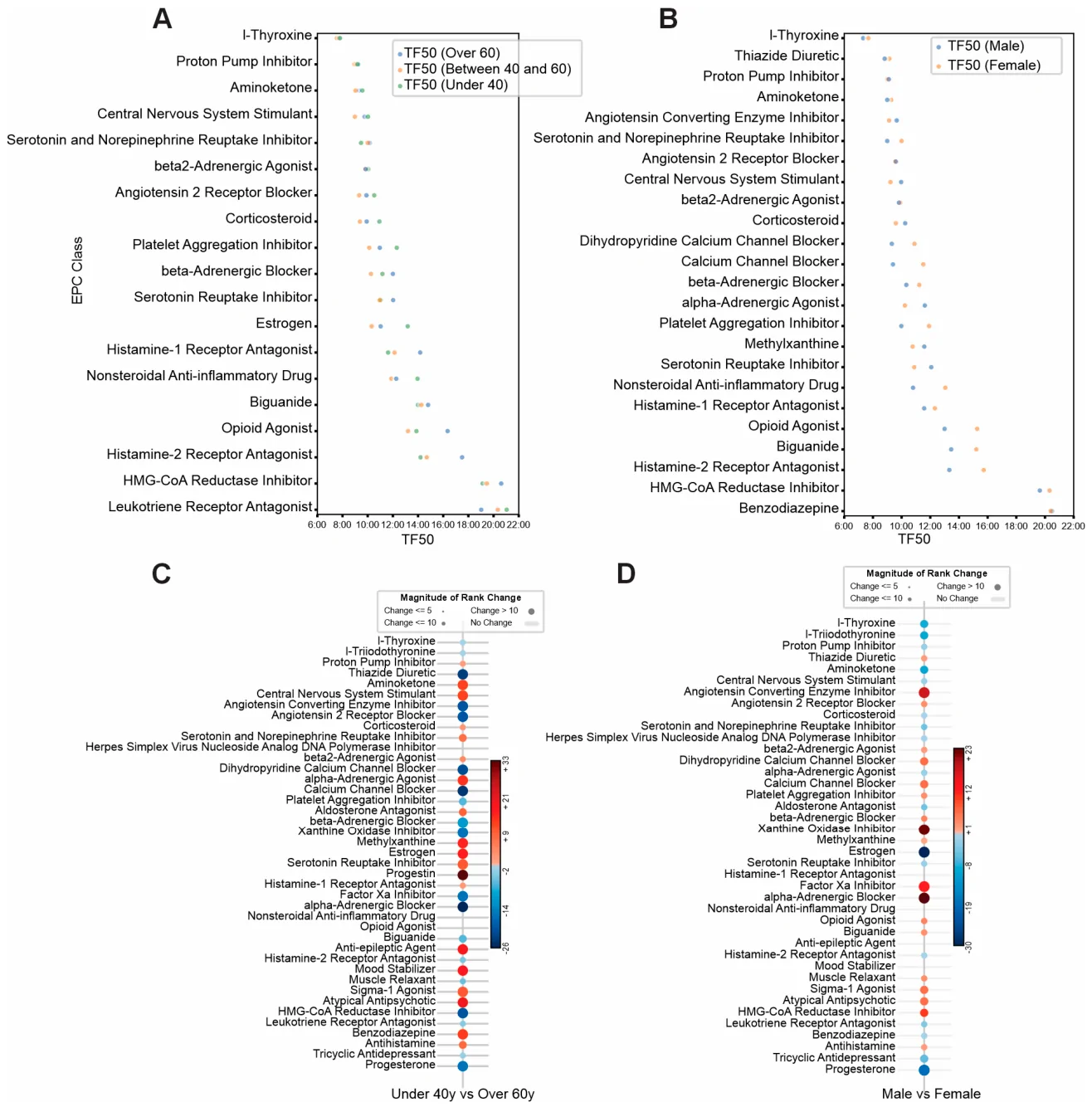
Age and sex influence the timing and relative prevalence of medication class intake. (**A**) The median time of intake (TF50) for the most frequently reported Established Pharmacologic Class (EPC) medications in males and females. TF50 represents the clock time at which 50% of all intake events for a given medication class have occurred. Most medication classes exhibit similar median intake times between sexes, although modest sex-specific differences are observed for few cardiovascular, neuropsychiatric, and anti-inflammatory medications. (**B**) TF50 for EPC medication classes stratified by age (<40 years, 40–60 years, and >60 years). While the overall temporal ordering of medication intake is largely preserved across age groups, several medication classes display age-dependent shifts in median dosing time, particularly among evening-administered medications. (**C**) Change in rank order of EPC medication classes between younger (<40 years) and older (>60 years) adults. Rank changes were calculated based on the relative proportion of the age group logging the EPC at least once. Circle color indicates the direction and magnitude of rank change (red, higher numeric rank or less intake in older adults; blue, higher numeric rank or less intake in younger adults), while circle size represents the magnitude of the rank shift. (**D**) The change in rank order of EPC medication classes between males and females. Red circle color implies higher numeric rank order or less use among females and blue implies less use among males.

We next examined whether medication timing and the relative prevalence of medication classes differed according to age and sex. To quantify timing independent of differences in the shape of the intake distributions, we calculated the TF50, defined as the clock time at which 50% of all intake events for a medication class had occurred. Across the 40 most frequently reported EPCs, TF50 values were remarkably consistent between males and females (Figure 3A, Table S8). Most morning medications—including L-thyroxine, proton pump inhibitors, thiazide diuretics, aminoketones, central nervous system stimulants, and serotonin-norepinephrine reuptake inhibitors—exhibited comparable median intake times in both sexes, typically between 07:00 and 10:00. Likewise, evening medications such as HMG-CoA reductase inhibitors and benzodiazepines retained similar TF50 values regardless of sex. Age exerted a modest influence on medication timing (Figure 3B, Table S9). Although the overall temporal ordering of medication classes remained highly conserved, several medication classes, particularly those taken during the afternoon or evening, showed shifts of approximately 1–2 h between younger (<40 years) and older (>60 years) adults, suggesting that medication timing is influenced by age-related differences in daily routines while remaining broadly aligned with therapeutic recommendations.

To quantify overall differences in the relative prevalence of medication classes between groups, we calculated Jensen-Shannon distance (JSD), which measures the dissimilarity between two probability distributions. Subgroup-level distributions were defined by the percentage of users in each subgroup reporting each EPC. The age group comparison produced a larger JSD than the comparison between sexes (age: JSD = 0.346; sex JSD = 0.256), indicating that age was associated with greater overall differences in medication class reporting patterns. Both observed distances exceeded those obtained under permutation testing age: mean permuted JSD = 0.021, maximum = 0.029; sex: mean = 0.029, maximum = 0.036; both *p* = 0.001). Because JSD provides a global summary of distributional dissimilarity but does not identify the medication classes responsible for these differences, we next examined subgroup-specific prevalence and changes in rank order (Figure 3C,D; Tables S10–S13).

Although the timing of medication intake was largely preserved across groups, the relative prevalence of medication classes changed with age (Figure 3C). Across all age groups, nonsteroidal anti-inflammatory drugs (NSAIDs) remained the most commonly used medication class, increasing in prevalence from 24.9% in adults < 40 years to 34.4% in adults > 60 years. In this cohort, younger adults reported more use of medications for mental health and attention disorders, including serotonin reuptake inhibitors (15.9%), central nervous system stimulants (12.3%), and aminoketones (5.8%), whereas older adults showed greater use of medications targeting chronic cardiometabolic disease, including HMG-CoA reductase inhibitors (20.5%), platelet aggregation inhibitors (18.8%), angiotensin II receptor blockers (13.5%), beta-adrenergic blockers (11.0%), and biguanides (9.3%) (Table S10). Thyroid hormone use also increased with age, rising from 13.3% in younger adults to 20.8% among older adults. These age-related differences were reflected by large shifts in medication ranking, with several medication classes changing by more than 20 positions (Figure 3C, Table S11).

Sex differences in medication use were relatively smaller than those associated with age, consistent with the lower JSD for the sex comparison (Figure 3D). The overall ranking of medication classes was comparable between males and females (Tables S12 and S13), with relatively few classes exhibiting larger shifts in rank order. NSAIDs remained the most frequently reported medication class in both sexes (29.8% in males and 27.8% in females). Males more frequently reported medications commonly prescribed for cardiovascular and metabolic disease, including HMG-CoA reductase inhibitors, platelet aggregation inhibitors, angiotensin II receptor blockers, beta blockers, and biguanides, whereas females showed relatively greater use of L-thyroxine, serotonin reuptake inhibitors, central nervous system stimulants, aminoketones, and estrogens. Correspondingly, the majority of rank changes between sexes were fewer than ten positions (Figure 3D), emphasizing that sex contributes less to medication selection than age.

A similar demographic pattern was observed for dietary supplements (Tables S14–S17), although differences were less pronounced than for medications. JSDs for supplement prevalence were 0.153 across age groups and 0.141 between males and females, both exceeding chance expectations in permutation testing (age: mean permuted JSD = 0.019, maximum = 0.027; sex: mean = 0.014, maximum = 0.017; both *p* = 0.001). The smaller JSDs for supplements than for EPC indicated that supplement popularity was more stable across demographic groups.

The most commonly consumed supplements—including multivitamins, vitamin D, magnesium, fish oil/omega-3, probiotics, and vitamin C—remained highly ranked across all age groups and in both sexes (Tables S14–S17). Older adults showed relatively greater use of supplements associated with healthy aging and musculoskeletal health, including calcium, vitamin B12, vitamin D3, and coenzyme Q10, whereas younger adults more frequently consumed probiotics and fish oil. Men exhibited modestly greater use of fish oil, omega-3, vitamin D3, and zinc, while women more commonly consumed magnesium, calcium, probiotics, vitamin C, and vitamin B12. Compared with medications, however, supplement rankings were considerably more stable across demographic groups (Tables S16 and S17), suggesting that supplement use is driven more by general health practices than by age- or sex-specific factors.

## 4. Discussion

Chronotherapy has traditionally sought to identify the optimal time to administer individual medications under controlled experimental conditions [16,17,18,45]. Whether patients take those medications at the optimal times in everyday life, however, has remained largely unknown. Using a large, real-world dataset of time-stamped medication and supplement intake, we constructed a population-scale atlas of intake behavior in free-living adults. Medication intake was highly organized rather than randomly distributed across the day. Medication timing was also substantially more stable across age and sex than the types of medications consumed. These real-world intake patterns offer a benchmark for chronotherapy implementation.

A central observation was the extent to which medication intake was anchored to common recurring daily behaviors and often consistent with established clinical guidance. Thyroid hormone, proton pump inhibitors, thiazide diuretics, corticosteroids, stimulants, and several antidepressant classes were concentrated in the first few hours after awakening. Conversely, statins, progesterone, benzodiazepines, antihistamines, and other evening-preferred medications clustered toward the end of the waking day. Re-expressing intake relative to wake time and bedtime strengthened these relationships. Specifically, the peak time of intake of Thyroid hormones within <2 h after awakening is consistent with the common recommendation for taking it in empty stomach after waking up. Therefore, medication schedules are frequently organized around awakening, meals, and sleep rather than around clock time alone. This distinction is important because the same clock hour can represent very different biological times for people with different sleep schedules, chronotypes, occupations, or social routines [46]. In contrast, metformin, beta-adrenergic blockers, calcium channel blockers, nonsteroidal anti-inflammatory drugs, and selective serotonin reuptake inhibitors showed broader or bimodal distributions. Such patterns may reflect twice-daily regimens, flexible instructions, administration with meals, symptom-triggered use, or individualized prescribing. For example, opioid use was more broadly distributed throughout the day, likely reflecting symptom related use. These classes may therefore require more nuanced chronotherapy strategies than a uniform recommendation to take the medication in the morning or evening.

Importantly, the prominent population peaks did not capture the full range of medication-taking behavior. Even for medications with highly synchronized intake, a small but notable proportion of doses occurred several hours away from the dominant period. For example, although levothyroxine was concentrated near awakening, some users took it much later in the day. This apparent “mistimed” dosing should not be interpreted as sub-optimal, because studies have shown levothyroxine taken at bedtime is also equally effective [47]. Similarly, medications with strong evening or bedtime peaks—including progesterone, rosuvastatin, benzodiazepines, and other sleep-associated therapies—were sometimes taken well before sleep onset. These extended tails are biologically and clinically meaningful because they show that real-world medication timing is a distribution rather than a single categorical label such as ‘morning’ or ‘bedtime.’ The observed dispersion may arise from clinician-directed alternative regimens, irregular work or sleep schedules, meal timing, caregiver routines, symptom onset, convenience, or incomplete adherence to instructions. Irrespective of cause, such temporal heterogeneity could contribute to variability in drug exposure, therapeutic response, and adverse effects, particularly for short-acting drugs or drugs whose targets, transporters, or metabolizing enzymes show large circadian oscillations.

This observation also broadens the concept of adherence. Conventional adherence measures focus on whether the correct dose was taken, often without considering its timing. Our findings suggest that temporal alignment may represent an additional dimension of medication adherence. A patient may be adherent by dose count yet repeatedly take a medication outside the intended physiological window. Conversely, a noncanonical dosing time may be intentional and clinically appropriate for that individual. Thus, the tails of the timing distributions should not automatically be interpreted as nonadherence; rather, they identify subgroups whose dosing schedules warrant contextual interpretation. Future chronotherapy studies should distinguish dose adherence from temporal adherence and determine whether deviations from an intended dosing window are associated with altered pharmacokinetics, efficacy, toxicity, or clinical outcomes. Such studies should also assess whether optimizing medication timing could achieve the same therapeutic benefit with lower doses.

Dietary supplements showed greater temporal diversity than medications. Most vitamins, minerals, and nutritional supplements were concentrated in the morning or around meals, but their degree of synchronization varied. Vitamin D, vitamin B12, multivitamins, probiotics, and coenzyme Q10 were morning-biased; fish oil, collagen, zinc, and curcumin were distributed more broadly across the day; magnesium showed both morning and evening use; and melatonin was tightly aligned with bedtime. These patterns likely reflect a combination of intended physiological effects, consumer guidance, meal-associated absorption or tolerability, and habitual routines. The contrast between melatonin and most nutritional supplements illustrates how intended function can strongly organize intake timing.

Age had a much greater influence on which medications were taken than on when they were taken. At least in this cohort, younger adults more frequently used medications for neuropsychiatric and attention-related conditions, whereas older adults more commonly used therapies for cardiovascular, metabolic, and other chronic diseases. Nevertheless, the temporal ordering of medication classes was broadly preserved across age groups. Sex differences in timing were also modest despite differences in the prevalence of selected medication classes. This conservation suggests that once a medication is incorporated into a daily regimen, its timing may be governed more by stable behavioral routines and dosing conventions than by demographic characteristics. It also implies that circadian optimization may be feasible without redesigning the entire medication routine: for selected drugs, relatively targeted shifts in timing may be sufficient.

These findings also point to practical opportunities for digital health. Smartphone applications and wearable devices can jointly capture medication intake, sleep, activity, light exposure, and meal timing. Such systems would need to preserve clinical context and avoid assuming that all deviations are inappropriate. Nevertheless, combining population timing norms with individualized behavioral data could make medication timing an actionable component of precision medicine alongside drug selection and dose.

## 5. Conclusions

This study provides a large-scale description of real-world medication and supplement timing and reveals three central principles. First, medication intake is structured around the beginning and end of the waking day, whereas supplements show additional meal-associated and function-specific patterns. Second, age and sex influence the composition of medication use far more than the timing of intake. Third, and critically, each dominant timing pattern includes a meaningful off-peak tail: a minority of users take even strongly morning- or bedtime-associated medications several hours away from awakening or sleep. Medication timing should therefore be viewed as a continuous behavioral phenotype with characteristic peaks, spread, and tails—not simply as a binary morning-versus-evening choice.

The major translational implication is that chronotherapy may not be implemented solely by determining when a medication should be taken; it must also account for when patients are able and willing to take it within their daily lives. Population timing distributions provide a benchmark for identifying temporal heterogeneity, but individualized biological time and clinical context will be essential for interpretation. As circadian precision medicine advances, assessment of temporal adherence should complement conventional dose adherence, and future trials should test whether aligning medication intake with both drug-specific biology and patient-specific sleep-wake schedules improves efficacy, safety, and long-term outcomes.

## 6. Limitations

Several limitations should be considered. First, medication and supplement intake was self-reported through a smartphone application and was not objectively confirmed. Although this study incorporated data quality procedures and sensitivity analyses, some entries may have been delayed, omitted, or retrospectively reconstructed. Second, the application captured reported intake time but not the original prescription instructions, dose, formulation, route, dosing frequency, treatment indication, or prescriber rationale. We therefore could not determine whether an apparently off-peak intake represented nonadherence, a clinician-directed alternative schedule, a different formulation, multiple daily dosing, or an intentional adaptation to the participant’s lifestyle. This is particularly important when interpreting the substantial tails surrounding otherwise well-defined morning and bedtime peaks.

Third, the cohort was self-selected and restricted to adults willing and able to use a digital health application. Participants may have been more health-conscious, technologically engaged, or motivated to record behaviors than the broader population, and geographic, socioeconomic, occupational, and racial or ethnic representation may not match that of the general population. Fourth, the primary observation window was limited to the first 14 days of app use. This interval was selected to capture habitual behavior before feedback could alter routines, but it may not reflect seasonal changes, intermittent medication use, evolving prescriptions, or long-term adherence. Fifth, clock time and self-reported sleep timing provide useful behavioral anchors but do not directly measure internal circadian phase; participants with shift work, jet lag, irregular sleep, or circadian disruption may therefore have been misclassified relative to biological time.

Finally, this observational analysis cannot determine whether the observed timing patterns are optimal or whether off-peak intake causes differences in efficacy, toxicity, or clinical outcomes. Nor can the data establish whether tighter temporal consistency would be beneficial for medications with broad or bimodal distributions. Future studies should integrate pharmacy and prescribing records, electronic adherence monitoring, dose and formulation information, validated circadian-phase measures, pharmacokinetic sampling, and longitudinal clinical outcomes. Randomized trials will ultimately be required to determine whether shifting medication timing toward drug- and patient-specific biological windows improves health beyond standard care.

## Data Availability

The datasets presented in this article are not readily available because of privacy concerns.

## Supplementary Materials

The following supporting information can be downloaded at: https://www.mdpi.com/article/10.3390/nu18193291/s1, Figure S1. Distribution of the number of medications and dietary supplements reported per participant. (A) Distribution of the number of unique medications logged per participant during the first 14 days of app use. More than half of participants reported taking a single medication, with progressively fewer participants reporting larger numbers of medications. Approximately 75% of participants reported one or two medications, whereas fewer than 8% reported five or more medications. (B) Distribution of the number of distinct dietary supplements logged per participant during the same period. Approximately 57% of participants logged one or two supplements, and a small fraction reported taking more than ten supplements. Figure S2. Daily timing of medication intake across major EPC classes. Histograms show the distribution of intake times for each Established Pharmacologic Class (EPC) across a 24-hour period using 1-hour bins. The blue bars represent the percentage of all intake events occurring within each hourly interval (left y-axis). The orange curve shows the cumulative percentage of daily intake (right y-axis). Vertical dashed lines and labels indicate the times by which 25%, 50%, and 75% of all daily intake events for each EPC class have occurred, illustrating differences in the temporal distribution of medication use across therapeutic classes. Panels are ordered by temporal intake pattern of EPC class, with each panel independently scaled on the y-axis to facilitate visualization of the distribution shape. Figure S3. Distribution of intake times for the most frequently reported individual medications. Each panel depicts the daily timing profile of a single medication, with blue histograms showing the percentage of intake events occurring within successive 1-hour intervals (left y-axis). The orange curve represents the cumulative proportion of total daily intake (right y-axis), highlighting how intake accumulates over the course of the day. The labeled markers identify the clock times at which 25%, 50%, and 75% of all reported doses for each medication had been consumed, providing a concise summary of the medication’s temporal intake profile. Panels are shown on independent y-axis scales to emphasize differences in the shape and concentration of intake patterns rather than absolute frequency. Figure S4. Daily intake profiles for the most commonly reported dietary supplements. For each supplement, the histogram displays the proportion of intake events recorded during each 1-hour interval throughout the day (left y-axis). The overlaid orange curve depicts the cumulative percentage of daily intake (right y-axis), illustrating the progression of supplement use from morning to night. Dashed vertical lines mark the times at which the cumulative intake reaches 25%, 50%, and 75% of all recorded doses, allowing comparison of the temporal distribution of supplement consumption across products. Individual panels use independent y-axis scales to better visualize differences in the concentration and timing of intake. Table S1. Participant and data summary. Table S2. EPC classes and their consumption time. Table S3. Example Medications and their time of consumption. Table S4. Medication timing relative to wake up time. Table S5. Medication timing relative to bedtime. Table S6. Supplements and their timing of consumption. Table S7. Supplement intake time relative to bedtime. Table S8. EPC classes and their TF50 in males and. females. Table S9. EPC classes and their TF50 in different age groups. Table S10. Rank order of EPC classes in young and old subgroups. Table S11. Change in rank order of EPC classes between young and old subgroups. Table S12. Rank order of medications in males and females Table S13. Change in rank order of EPC classes between males and females. Table S14. Rank order of supplements among males and females. Table S15. Rank order of supplements among different age groups. Table S16. Change in rank order of supplements between young and old subgroups. Table S17. Change in rank order of supplements between males and females. Section S1. Supplementary text on Prompt usage.
