# Supplementary material for: Daily Patterns in the Timing of Medication and Supplement Intake
Source: Nutrients. 2026 Oct 7;18(19):3291. doi: 10.3390/nu18193291 (PMC13667124; doi:10.3390/nu18193291)
Supplement: Supplementary file 1 [file nutrients-18-03291-s001.zip › nutrients-4530331-supplementary.pdf]

Daily Patterns in the Timing of Medication and Supplement Intake

Tyler Tran[1,*], Shweta Varshney[1,*], Laura van Rosmalen[1], Jordan L. Levy[1], Kyla L. Laing[1], Emily N. C. Manoogian[1], Michael Tun Yin Lam [2], Satchidananda Panda[1,3]

Supplemental Materials.

Supplementary Figures and figure legends S1–S4
Supplementary Tables S1–S17
Section S1. Supplementary text on Prompt usage.

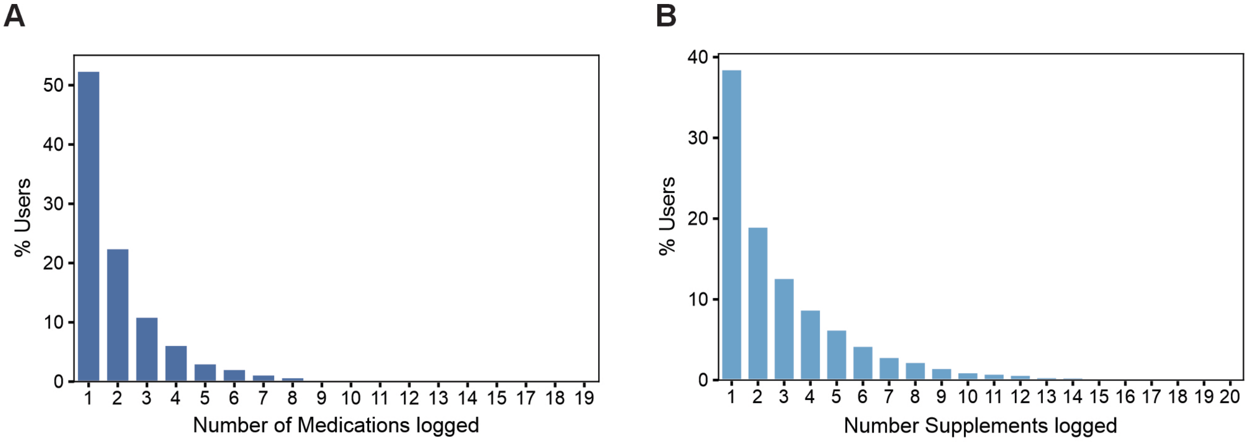

Figure S1. Distribution of the number of medications and dietary supplements reported per participant. (A) Distribution of the number of unique medications logged per participant during the first 14 days of app use. More than half of participants reported taking a single medication, with progressively fewer participants reporting larger numbers of medications. Approximately 75% of participants reported one or two medications, whereas fewer than 8% reported five or more medications. **(B)** Distribution of the number of distinct dietary supplements logged per participant during the same period. Approximately 57% of participants logged one or two supplements, and a small fraction reported taking more than ten supplements.

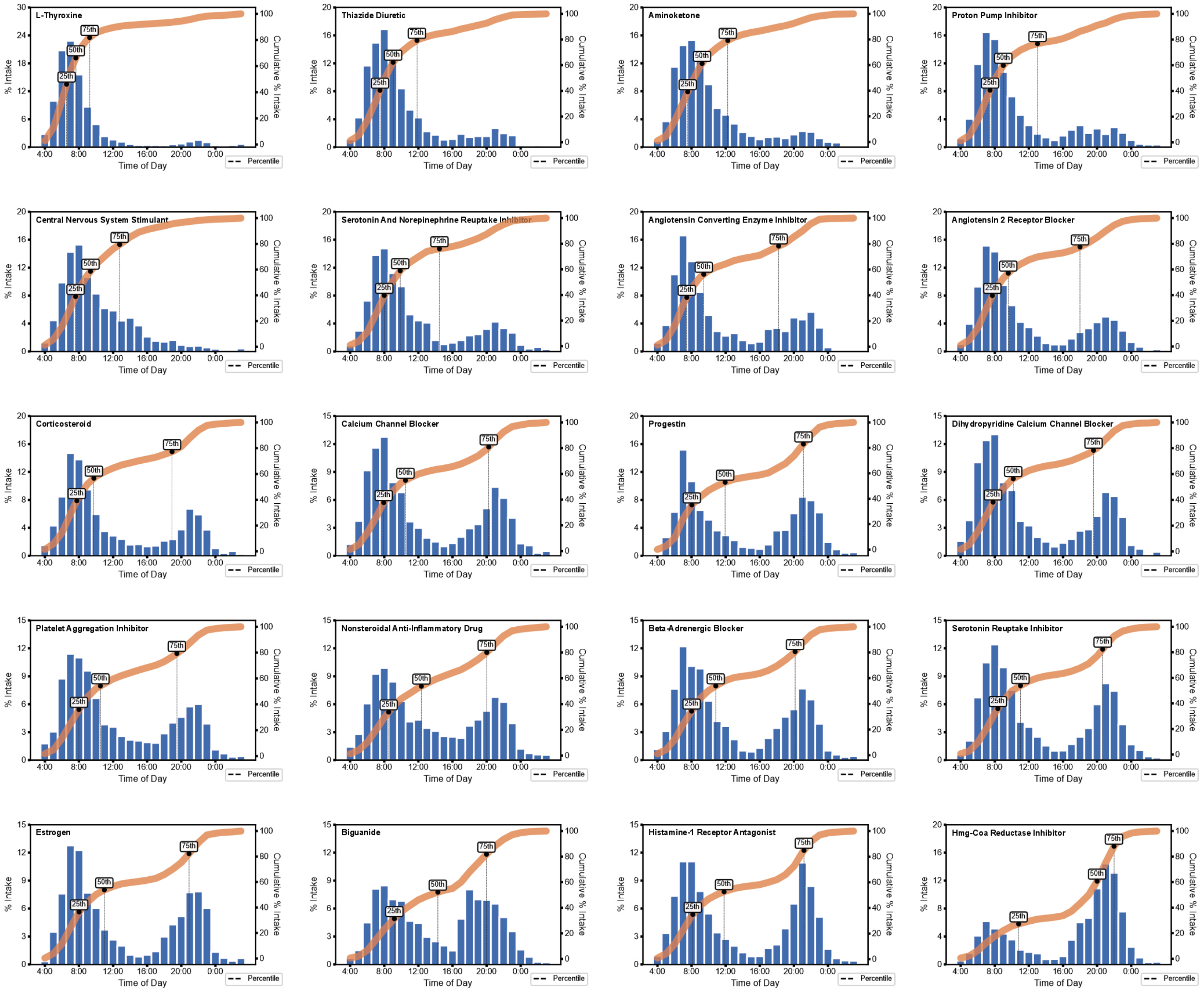

Figure S2. Daily timing of medication intake across major EPC classes. Histograms show the distribution of intake times for each Established Pharmacologic Class (EPC) across a 24-hour period using 1-hour bins. The blue bars represent the percentage of all intake events occurring within each hourly interval (left y-axis). The orange curve shows the cumulative percentage of daily intake (right y-axis). Vertical dashed lines and labels indicate the times by which 25%, 50%, and 75% of all daily intake events for each EPC class have occurred, illustrating differences in the temporal distribution of medication use across therapeutic classes. Panels are ordered by temporal intake pattern of EPC class, with each panel independently scaled on the y-axis to facilitate visualization of the distribution shape.

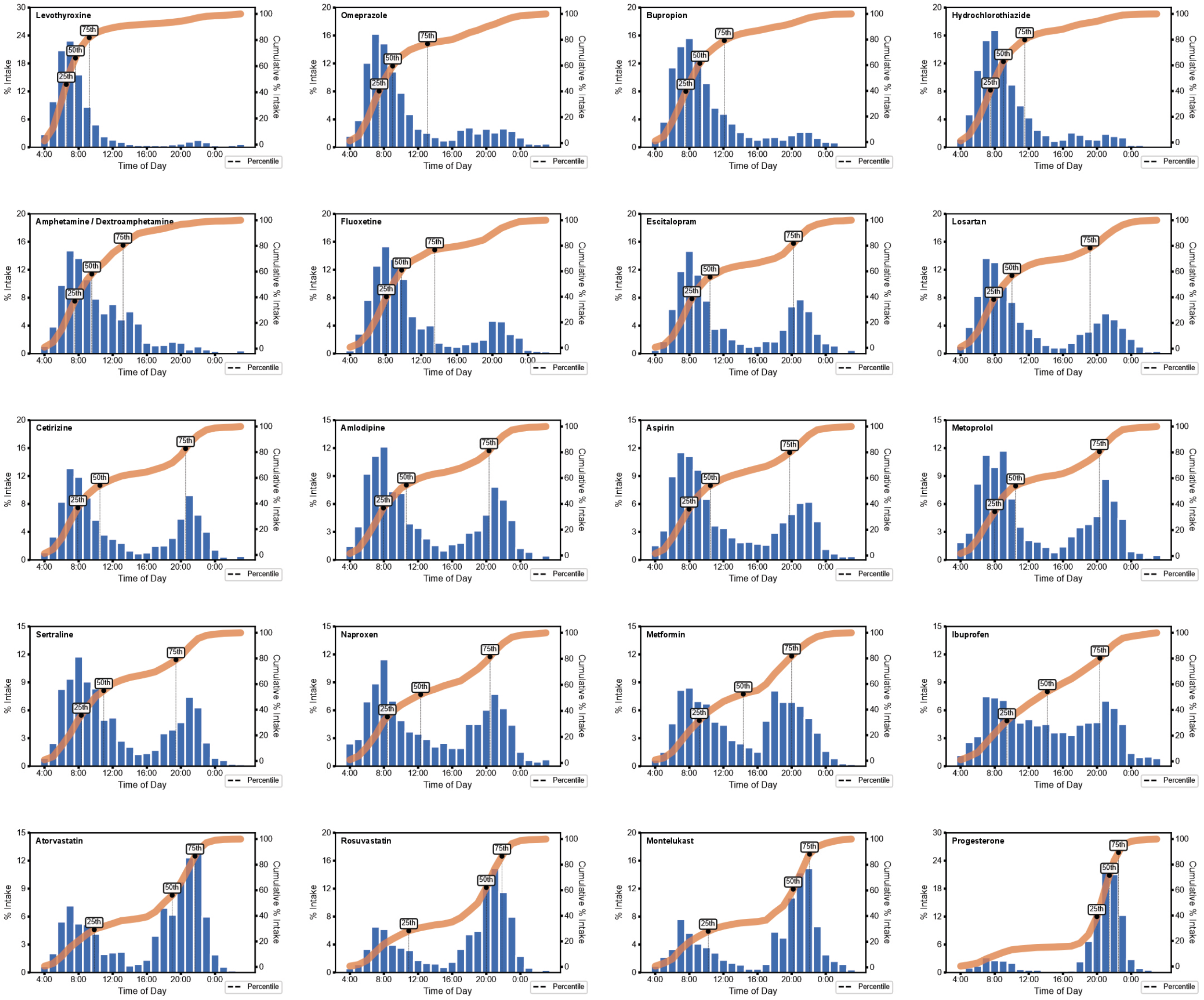

Figure S3. Distribution of intake times for the most frequently reported individual medications. Each panel depicts the daily timing profile of a single medication, with blue histograms showing the percentage of intake events occurring within successive 1-hour intervals (left y-axis). The orange curve represents the cumulative proportion of total daily intake (right y-axis), highlighting how intake accumulates over the course of the day. The labeled markers identify the clock times at which 25%, 50%, and 75% of all reported doses for each medication had been consumed, providing a concise summary of the medication's temporal intake profile. Panels are shown on independent y-axis scales to emphasize differences in the shape and concentration of intake patterns rather than absolute frequency.

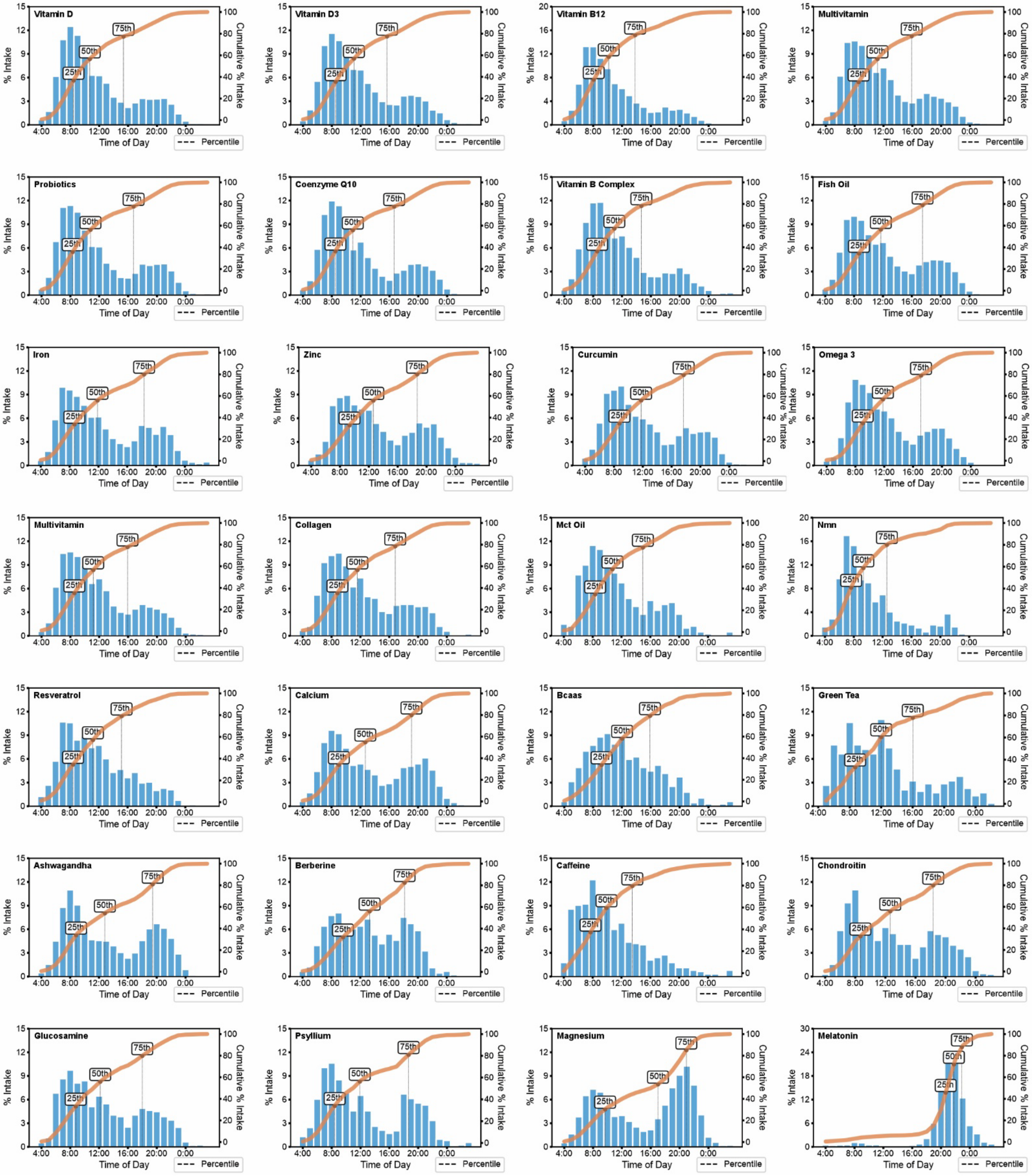

Figure S4: Daily intake profiles for the most commonly reported dietary supplements. For each supplement, the histogram displays the proportion of intake events recorded during each 1-hour interval throughout the day (left y-axis). The overlaid orange curve depicts the cumulative percentage of daily intake (right y-axis), illustrating the progression of supplement use from morning to night. Dashed vertical lines mark the times at which the cumulative intake reaches 25%, 50%, and 75% of all recorded

doses, allowing comparison of the temporal distribution of supplement consumption across products. Individual panels use independent y-axis scales to better visualize differences in the concentration and timing of intake.

Supplemental Table S1

Participant and data summary

	Male	Female	Unknown	Total
Total Participants	8276	17743	50	26069
**Age**				
<40 yrs	3341	6054	34	9429
40-60 yrs	3956	9689	4	13649
>60 yrs	979	2000	2	2981
Unknown	0	0	10	10
**Medications**				
Total number of medications analyzed	37356	89406	267	127029
Total number of supplements analyzed	77438	170403	329	248170
Number of medications <40 y old	7684	17876	177	25737
Number of medications 40-60 y old	19375	54364	20	73759
Number of medications >60 y old	10297	17166	4	27467
Number of Supplements <40 y old	30473	48127	204	78804
Number of Supplements 40-60 y old	37224	95895	11	133130
Number of supplements >60 y old	9741	26381	0	36122
**Geographic Location**				
United States	4131	9523	19	13673

	Male	Female	Unknown	Total
United Kingdom	499	1314	1	1814
Other	3204	5854	30	9088
Unknown	442	1052	0	1494

Supplemental Table S2

EPC classes and their consumption time

rank	epc_class	tf50	tf25	tf75	dip	p
1	Nonsteroidal Anti-inflammatory Drug	12.31	8.52	20.02	0.03	0.0000
2	l-Thyroxine	7.63	6.55	9.25	0.01	0.0000
3	Serotonin Reuptake Inhibitor	11.06	8.37	20.69	0.06	0.0000
4	Histamine-1 Receptor Antagonist	12.00	8.18	21.22	0.08	0.0000
5	Platelet Aggregation Inhibitor	10.52	8.00	19.48	0.04	0.0000
6	Central Nervous System Stimulant	9.43	7.59	12.88	0.01	0.0491
7	HMG-CoA Reductase Inhibitor	20.00	10.81	21.98	0.05	0.0000
8	Proton Pump Inhibitor	9.01	7.45	13.08	0.01	0.0000
9	Angiotensin 2 Receptor Blocker	9.55	7.72	18.00	0.03	0.0000
10	Biguanide	14.28	9.18	19.97	0.06	0.0000
11	beta-Adrenergic Blocker	10.82	8.00	20.10	0.05	0.0000
12	Aminoketone	9.23	7.55	12.25	0.01	0.2688
13	Corticosteroid	9.80	7.75	19.58	0.05	0.0000
14	Calcium Channel Blocker	10.46	7.93	20.24	0.05	0.0000
15	Dihydropyridine Calcium Channel Blocker	10.15	7.80	19.58	0.05	0.0000
16	Estrogen	10.99	8.02	20.92	0.07	0.0000
17	Serotonin and Norepinephrine Reuptake Inhibitor	9.86	8.00	14.46	0.02	0.0000
18	Thiazide Diuretic	9.03	7.50	11.83	0.01	0.1499
19	Progestin	11.94	8.04	21.12	0.07	0.0000
20	Benzodiazepine	20.45	11.60	22.60	0.03	0.0000

rank	epc_class	tf50	tf25	tf75	dip	p
21	Methylxanthine	10.93	8.12	15.74	0.01	0.9827
22	Progesterone	21.44	19.88	22.51	0.03	0.0000
23	alpha-Adrenergic Agonist	10.43	8.02	18.67	0.03	0.0000
24	Angiotensin Converting Enzyme Inhibitor	9.43	7.43	18.21	0.04	0.0000
25	beta2-Adrenergic Agonist	9.88	7.70	20.75	0.07	0.0000
26	Histamine-2 Receptor Antagonist	14.78	8.99	21.25	0.06	0.0000
27	Leukotriene Receptor Antagonist	20.17	10.19	22.10	0.06	0.0000
28	Opioid Agonist	14.11	9.04	19.38	0.03	0.0000
29	Mood Stabilizer	15.02	9.23	21.50	0.09	0.0000
30	Anti-epileptic Agent	14.72	9.12	21.50	0.09	0.0000
31	Tricyclic Antidepressant	21.13	19.85	22.50	0.02	0.1472
32	alpha-Adrenergic Blocker	12.21	8.30	20.91	0.08	0.0000
33	l-Triiodothyronine	7.99	6.48	10.61	0.01	0.8214
34	Aldosterone Antagonist	10.60	7.96	19.68	0.05	0.0000
35	Atypical Antipsychotic	20.00	11.23	21.93	0.06	0.0000
36	Antihistamine	20.92	13.58	22.48	0.04	0.0001
37	Sigma-1 Agonist	19.44	11.30	22.22	0.03	0.0702
40	Herpes Simplex Virus Nucleoside Analog DNA Polymerase Inhibitor	9.88	8.02	19.01	0.05	0.0000
42	Muscle Relaxant	19.27	11.88	22.17	0.03	0.0018
43	Xanthine Oxidase Inhibitor	10.87	7.95	18.56	0.04	0.0000
44	Factor Xa Inhibitor	12.18	8.40	19.98	0.09	0.0000

Supplemental Table S3

Example Medications and their time of consumption

rank		tf50	tf25	tf75	dip	p
1	levothyroxine	7.63	6.55	9.25	0.01	0.0000
2	ibuprofen	14.17	9.43	20.33	0.02	0.0000
3	aspirin	10.47	7.97	19.77	0.04	0.0000
4	metformin	14.33	9.17	20.00	0.06	0.0000
5	cetirizine	10.50	7.92	20.55	0.06	0.0000
6	bupropion	9.23	7.58	12.10	0.01	0.2209
7	atorvastatin	18.97	9.83	21.65	0.06	0.0000
8	sertraline	10.95	8.32	19.42	0.04	0.0000
9	omeprazole	9.02	7.43	13.11	0.02	0.0013
10	naproxen	12.30	8.40	20.44	0.05	0.0000
11	losartan	10.02	7.88	19.18	0.04	0.0000
12	amlodipine	10.63	7.93	20.32	0.05	0.0000
13	escitalopram	10.43	8.30	20.22	0.06	0.0000
14	fluoxetine	9.80	8.08	13.52	0.03	0.0000
15	amphetamine / dextroamphetamine	9.55	7.55	13.21	0.01	0.6560
16	diphenhydramine	21.30	19.37	22.50	0.01	0.6541
17	hydrochlorothiazide	9.02	7.50	11.52	0.01	0.8630
18	metoprolol	10.45	8.00	20.28	0.06	0.0000
19	progesterone	21.45	19.99	22.52	0.03	0.0000
20	rosuvastatin	20.00	10.91	21.84	0.04	0.0000

Supplemental Table S4

Medication timing relative to wake up time

medication	avg_wake_to_intake_hours	median_wake_to_intake_hours	sd_wake_to_intake_hours	n_logs	n_users
levothyroxine	1.14	0.53	1.72	5554	808
aspirin	3.23	2.08	3.17	1516	321
metformin	4.86	3.50	3.97	1494	206
ibuprofen	4.86	4.10	3.59	1457	667
bupropion	2.43	1.42	2.67	1337	187
cetirizine	2.60	1.57	2.92	1159	249
omeprazole	2.43	1.36	2.85	974	168
losartan	2.57	1.50	2.91	846	139
hydrochlorothiazide	2.42	1.62	2.53	819	122
amlodipine	2.64	1.35	3.12	779	128
sertraline	3.19	1.81	3.24	744	130
fluoxetine	2.57	1.78	2.44	607	103
escitalopram	2.66	1.67	2.81	585	107
naproxen	4.08	2.72	3.66	479	165

Supplemental Table S5

Medication timing relative to bed time

medication	avg_intake_to_bed_hours	median_intake_to_bed_hours	sd_intake_to_bed_hours	n_logs	n_users
atorvastatin	2.44	1.33	2.83	824	167
progesterone	1.28	0.79	1.77	586	122
rosuvastatin	2.55	1.40	2.89	387	84
montelukast	2.27	1.26	2.71	340	79
amitriptyline	1.71	1.22	1.76	329	73
simvastatin	1.64	0.88	1.93	294	52
clonazepam	2.53	1.50	3.02	133	30
cyclobenzaprine	1.55	0.72	2.29	96	30
acetaminophen / dextromethorphan / doxylamine	1.76	1.04	2.48	58	35

Supplemental Table S6

Supplements and their timing of consumption

rank		tf50	tf25	tf75	dip	p
1	multivitamin	11.17	8.55	15.92	0.01	0.0001
2	vitamin d	10.68	8.43	15.37	0.01	0.0000
3	magnesium	17.00	9.67	20.93	0.05	0.0000
4	vitamin c	11.28	8.63	16.33	0.01	0.0097
5	probiotics	10.78	8.32	16.77	0.02	0.0000
6	vitamin	10.95	8.40	16.53	0.01	0.0000
7	vitamin d3	11.07	8.53	15.66	0.01	0.0001
8	fish oil	11.70	8.67	17.50	0.01	0.0000
9	omega 3	11.82	9.02	17.10	0.01	0.0000
10	vitamin b12	10.15	8.07	13.82	0.01	0.2782
11	calcium	12.68	9.02	19.08	0.02	0.0000
12	supplement	11.72	8.82	17.87	0.02	0.0000
13	zinc	12.62	9.32	18.70	0.02	0.0000
14	curcumin	11.77	8.75	17.67	0.01	0.0000
15	melatonin	21.78	20.65	22.90	0.01	0.1396
16	vitamin b complex	10.93	8.55	14.68	0.01	0.5057
17	iron	11.87	8.67	18.33	0.02	0.0000
18	coenzyme q10	10.92	8.50	16.66	0.01	0.0009
19	collagen	11.58	8.82	16.83	0.01	0.4873
20	vitamin k2	11.40	8.66	15.24	0.01	0.1219

rank		tf50	tf25	tf75	dip	p
21	vitamin b	10.00	8.30	13.40	0.01	0.9904
22	glucosamine	12.16	8.75	17.98	0.01	0.0141
23	biotin	11.38	8.58	17.08	0.01	0.0620
24	krill oil	11.16	8.42	15.81	0.01	0.3844
25	creatine	11.47	8.37	16.17	0.01	0.9389
26	ashwagandha	12.82	8.81	19.43	0.03	0.0000
27	vitamin e	11.96	9.02	17.41	0.01	0.0189
28	potassium	13.00	9.13	18.97	0.03	0.0000
29	vitamin b9	10.37	8.33	14.65	0.01	0.6435
30	cod liver oil	10.98	8.84	14.78	0.01	0.2283
31	psyllium	11.90	8.42	18.67	0.04	0.0000
32	fiber	11.90	8.67	18.38	0.03	0.0000
33	resveratrol	11.19	8.43	15.16	0.01	0.9665
34	digestive enzymes	13.50	10.41	18.42	0.02	0.0010
35	selenium	12.22	9.08	16.43	0.02	0.0900
36	vitamin k	10.54	8.70	14.32	0.01	0.9743
37	mushrooms	11.31	8.40	15.16	0.01	0.9479
38	spirulina	12.07	9.13	16.27	0.01	0.8465
39	iodine	11.03	8.50	15.43	0.01	0.6473
40	bcaas	12.02	9.17	15.89	0.01	0.8799
41	chondroitin	12.65	8.68	18.36	0.02	0.0086
42	vitamin b6	11.42	8.55	18.24	0.03	0.0003

rank		tf50	tf25	tf75	dip	p
43	5 htp	16.25	9.91	21.08	0.05	0.0000
44	milk thistle	11.93	9.12	18.38	0.03	0.0005
45	nac	12.15	8.85	18.13	0.02	0.0148
46	minerals	11.85	8.77	18.50	0.03	0.0000
47	msm	11.82	8.60	15.83	0.01	0.3487
48	apple cider vinegar	11.36	8.68	17.24	0.01	0.6415
49	garlic	12.84	9.02	19.43	0.02	0.0047
50	caffeine	9.92	7.70	13.50	0.01	0.9907
51	dhea	9.49	7.69	12.91	0.02	0.1823
52	berberine	13.30	9.60	18.15	0.02	0.0196
53	ala	12.23	9.13	18.02	0.01	0.6945
54	l theanine	15.40	9.38	21.25	0.06	0.0000
55	vitamin a	10.99	8.78	15.61	0.02	0.0430
56	evening primrose oil	11.63	9.05	17.33	0.02	0.0521
57	electrolytes	11.85	8.60	17.38	0.01	0.8955
58	ginkgo biloba	10.48	8.18	15.70	0.02	0.4494
59	chromium	11.96	8.88	16.45	0.01	0.6505
60	green tea	12.00	8.69	16.01	0.02	0.3069
61	nmn	9.32	7.63	12.59	0.01	0.5942
62	lysine	11.69	9.00	18.29	0.04	0.0003
63	chlorella	11.77	8.67	17.78	0.03	0.0122
64	niacin	12.44	8.51	19.98	0.05	0.0000

rank		tf50	tf25	tf75	dip	p
65	mct oil	10.74	8.34	14.96	0.01	0.9572
66	flaxseed oil	11.42	8.58	17.99	0.02	0.0363

Supplemental Table S7

Supplement intake time relative to bed time

supplement	avg_intake_to_bed_hours	median_intake_to_bed_hours	sd_intake_to_bed_hours	n_logs	n_users
magnesium	3.69	2.32	3.56	5819	1480
multivitamin	6.55	7.07	3.81	3841	1109
vitamin d	6.40	7.05	3.92	3590	1113
omega 3	6.61	6.99	3.71	2612	734
vitamin c	6.35	6.71	3.81	2586	788
vitamin d3	6.75	7.45	3.80	2483	750
probiotics	5.96	5.38	3.98	2359	763
vitamin	6.23	6.40	3.98	1981	598
melatonin	1.08	0.77	1.25	1781	508
vitamin b12	7.14	8.05	3.62	1199	457
vitamin b complex	7.11	8.48	3.80	875	286
vitamin k2	6.95	7.83	3.79	770	219
coenzyme q10	6.11	6.00	4.05	736	210
collagen	6.37	6.72	3.83	618	198
krill oil	6.50	7.10	3.86	467	126
biotin	6.24	7.05	3.90	362	121

supplement	avg_intake_to_bed_hours	median_intake_to_bed_hours	sd_intake_to_bed_hours	n_logs	n_users
cod liver oil	6.67	7.11	3.50	359	104
vitamin b	7.40	8.35	3.51	302	129

Supplemental Table S8

EPC classes and their TF50 in males and females

epc_class	TF50 (Male)	TF50 (Female)
Aminoketone	9.00	9.28
Angiotensin 2 Receptor Blocker	9.60	9.55
Angiotensin Converting Enzyme Inhibitor	9.66	9.13
Benzodiazepine	20.50	20.40
Biguanide	13.46	15.21
Calcium Channel Blocker	9.40	11.51
Central Nervous System Stimulant	9.97	9.22
Corticosteroid	10.25	9.60
Dihydropyridine Calcium Channel Blocker	9.32	10.90
HMG-CoA Reductase Inhibitor	19.64	20.32
Histamine-1 Receptor Antagonist	11.58	12.32
Histamine-2 Receptor Antagonist	13.33	15.73
Methylxanthine	11.58	10.77
Nonsteroidal Anti-inflammatory Drug	10.80	13.05
Opioid Agonist	13.00	15.27
Platelet Aggregation Inhibitor	9.98	11.92
Proton Pump Inhibitor	9.11	9.00
Serotonin Reuptake Inhibitor	12.07	10.88
Serotonin and Norepinephrine Reuptake Inhibitor	9.00	10.00
Thiazide Diuretic	8.82	9.15

epc_class	TF50 (Male)	TF50 (Female)
alpha-Adrenergic Agonist	11.62	10.23
beta-Adrenergic Blocker	10.33	11.23
beta2-Adrenergic Agonist	9.82	9.92
l-Thyroxine	7.32	7.68

Supplemental Table S9

EPC classes and their TF50 in different age groups

epc_class	TF50 (Under 40)	TF50 (Between 40 and 60)	TF50 (Over 60)
Aminoketone	9.58	9.03	9.32
Angiotensin 2 Receptor Blocker	10.52	9.32	9.90
Biguanide	14.00	14.27	14.80
Central Nervous System Stimulant	10.03	8.98	9.76
Corticosteroid	10.93	9.38	9.92
Estrogen	13.18	10.31	11.03
HMG-CoA Reductase Inhibitor	19.13	19.47	20.61
Histamine-1 Receptor Antagonist	11.62	12.13	14.17
Histamine-2 Receptor Antagonist	14.20	14.69	17.50
Leukotriene Receptor Antagonist	21.04	20.34	19.02
Nonsteroidal Anti-inflammatory Drug	13.96	11.88	12.27
Opioid Agonist	13.87	13.20	16.35
Platelet Aggregation Inhibitor	12.31	10.12	10.96
Proton Pump Inhibitor	9.18	8.95	9.25
Serotonin Reuptake Inhibitor	11.03	10.95	12.02
Serotonin and Norepinephrine Reuptake Inhibitor	9.47	10.00	10.17
beta-Adrenergic Blocker	11.17	10.26	12.00
beta2-Adrenergic Agonist	10.05	9.84	9.83
l-Thyroxine	7.80	7.55	7.72

Supplemental Table S10

Rank order of EPC classes in young and old sub groups

rank	epc_class	percent_young	epc_class	percent_mid	epc_class	percent_old
1	Nonsteroidal Anti-inflammatory Drug	24.92	Nonsteroidal Anti-inflammatory Drug	28.56	Nonsteroidal Anti-inflammatory Drug	34.37
2	Serotonin Reuptake Inhibitor	15.85	l-Thyroxine	18.36	l-Thyroxine	20.84
3	l-Thyroxine	13.29	Histamine-1 Receptor Antagonist	12.07	HMG-CoA Reductase Inhibitor	20.48
4	Central Nervous System Stimulant	12.28	Serotonin Reuptake Inhibitor	11.87	Platelet Aggregation Inhibitor	18.83
5	Histamine-1 Receptor Antagonist	11.85	Platelet Aggregation Inhibitor	9.2	Angiotensin 2 Receptor Blocker	13.47
6	Aminoketone	5.83	HMG-CoA Reductase Inhibitor	7.61	beta-Adrenergic Blocker	10.97
7	Progestin	5.62	Proton Pump Inhibitor	7.36	Biguanide	9.26
8	Proton Pump Inhibitor	5.28	Angiotensin 2 Receptor Blocker	7.19	Histamine-1 Receptor Antagonist	8.84
9	Estrogen	4.95	Central Nervous System Stimulant	6.6	Calcium Channel Blocker	8.84

rank	epc_class	percent_young	epc_class	percent_mid	epc_class	percent_old
10	Platelet Aggregation Inhibitor	4.34	Biguanide	6.5	Proton Pump Inhibitor	8.71
11	Corticosteroid	3.97	beta-Adrenergic Blocker	5.71	Dihydropyridine Calcium Channel Blocker	7.92
12	Serotonin and Norepinephrine Reuptake Inhibitor	3.7	Aminoketone	5.18	Thiazide Diuretic	7.56
13	Biguanide	3.63	Corticosteroid	5.1	Serotonin Reuptake Inhibitor	7.5
14	Methylxanthine	2.87	Calcium Channel Blocker	4.87	Corticosteroid	5.42
15	Benzodiazepine	2.57	Dihydropyridine Calcium Channel Blocker	4.49	alpha-Adrenergic Blocker	4.14
16	beta-Adrenergic Blocker	2.29	Thiazide Diuretic	4.06	Angiotensin Converting Enzyme Inhibitor	3.78
17	Mood Stabilizer	2.29	Serotonin and Norepinephrine Reuptake Inhibitor	3.84	Central Nervous System Stimulant	3.66
18	Anti-epileptic Agent	2.26	Estrogen	3.68	Histamine-2 Receptor Antagonist	3.35
19	alpha-Adrenergic Agonist	2.11	Progesterone	2.87	Aminoketone	3.23

rank	epc_class	percent_young	epc_class	percent_mid	epc_class	percent_old
20	beta2-Adrenergic Agonist	1.74	Benzodiazepine	2.42	Serotonin and Norepinephrine Reuptake Inhibitor	3.11
21	Atypical Antipsychotic	1.71	alpha-Adrenergic Agonist	2.42	Leukotriene Receptor Antagonist	2.99
22	Histamine-2 Receptor Antagonist	1.59	Angiotensin Converting Enzyme Inhibitor	2.31	Progesterone	2.56
23	Angiotensin 2 Receptor Blocker	1.44	Opioid Agonist	2.2	Factor Xa Inhibitor	2.32
24	Leukotriene Receptor Antagonist	1.44	Methylxanthine	2.18	Xanthine Oxidase Inhibitor	2.19
25	HMG-CoA Reductase Inhibitor	1.44	beta2-Adrenergic Agonist	2.04	beta2-Adrenergic Agonist	2.13
26	Opioid Agonist	1.41	Leukotriene Receptor Antagonist	1.97	Estrogen	2.01
27	Aldosterone Antagonist	1.41	Histamine-2 Receptor Antagonist	1.79	Opioid Agonist	2.01
28	Sigma-1 Agonist	1.25	l-Triiodothyronine	1.67	Tricyclic Antidepressant	1.95
29	Antihistamine	1.13	Progestin	1.61	Sulfonylurea	1.71

rank	epc_class	percent_young	epc_class	percent_mid	epc_class	percent_old
30	Progestin-containing Intrauterine System	1.04	Tricyclic Antidepressant	1.58	P2Y12 Platelet Inhibitor	1.65
31	Tricyclic Antidepressant	1.01	Mood Stabilizer	1.45	Osmotic Laxative	1.58
32	Herpes Simplex Virus Nucleoside Analog DNA Polymerase Inhibitor	0.98	Anti-epileptic Agent	1.34	Benzodiazepine	1.58
33	Sympathomimetic-like Agent	0.98	Muscle Relaxant	1.2	Antiarrhythmic	1.52
34	Dihydropyridine Calcium Channel Blocker	0.98	alpha-Adrenergic Blocker	1.19	Methylxanthine	1.4
35	alpha-1 Adrenergic Agonist	0.95	Antihistamine	1.15	5-alpha Reductase Inhibitor	1.4
36	Calcium Channel Blocker	0.95	Aldosterone Antagonist	1.09	gamma-Aminobutyric Acid A Receptor Positive Modulator	1.28
37	l-Triiodothyronine	0.92	Sigma-1 Agonist	1.05	Herpes Simplex Virus Nucleoside Analog DNA Polymerase Inhibitor	1.28

rank	epc_class	percent_young	epc_class	percent_mid	epc_class	percent_old
38	Tetracycline-class Drug	0.89	Herpes Simplex Virus Nucleoside Analog DNA Polymerase Inhibitor	1	Muscle Relaxant	1.16
39	Aminosalicylate	0.86	Serotonin-1b and Serotonin-1d Receptor Agonist	0.97	Thiazide-like Diuretic	1.1
40	5-alpha Reductase Inhibitor	0.82	Xanthine Oxidase Inhibitor	0.94	l-Triiodothyronine	1.1
41	Expectorant	0.79	Atypical Antipsychotic	0.94	Aromatase Inhibitor	1.1
42	Cholinergic Nicotinic Agonist	0.7	Androgen	0.86	Dietary Cholesterol Absorption Inhibitor	1.04
43	Thiazide Diuretic	0.67	gamma-Aminobutyric Acid A Receptor Positive Modulator	0.84	Peroxisome Proliferator Receptor alpha Agonist	1.04
44	gamma-Aminobutyric Acid A Receptor Positive Modulator	0.61	Antirheumatic Agent	0.8	alpha-Adrenergic Agonist	1.04
45	Sympathomimetic Amine Anorectic	0.58	Factor Xa Inhibitor	0.8	Mood Stabilizer	0.98

rank	epc_class	percent_young	epc_class	percent_mid	epc_class	percent_old
46	Angiotensin Converting Enzyme Inhibitor	0.58	Expectorant	0.76	Potassium-sparing Diuretic	0.98
47	Retinoid	0.55	Tetracycline-class Drug	0.73	Phosphodiesterase 5 Inhibitor	0.91
48	Progesterone	0.52	Sympathomimetic Amine Anorectic	0.72	Aldosterone Antagonist	0.85
49	Osmotic Laxative	0.52	Insulin	0.7	Cholinergic Muscarinic Antagonist	0.85
50	Muscle Relaxant	0.52	Antimalarial	0.7	Androgen	0.79

Supplemental Table S11

Change in rank order of EPC classes between young and old subgroups

epc_class	rank_under 40	rank_over 60	rank_change
l-Thyroxine	3	2	-1
l-Triiodothyronine	34	33	-1
Proton Pump Inhibitor	8	10	2
Thiazide Diuretic	35	12	-23
Aminoketone	6	19	13
Central Nervous System Stimulant	4	17	13
Angiotensin Converting Enzyme Inhibitor	36	16	-20
Angiotensin 2 Receptor Blocker	25	5	-20
Corticosteroid	11	14	3
Serotonin and Norepinephrine Reuptake Inhibitor	12	20	8
Herpes Simplex Virus Nucleoside Analog DNA Polymerase Inhibitor	31	31	0
beta2-Adrenergic Agonist	20	25	5
Dihydropyridine Calcium Channel Blocker	32	11	-21
alpha-Adrenergic Agonist	19	34	15
Calcium Channel Blocker	33	9	-24
Platelet Aggregation Inhibitor	10	4	-6
Aldosterone Antagonist	26	36	10
beta-Adrenergic Blocker	17	6	-11
Xanthine Oxidase Inhibitor	40	24	-16

epc_class	rank_under 40	rank_over 60	rank_change
Methylxanthine	14	30	16
Estrogen	9	26	17
Serotonin Reuptake Inhibitor	2	13	11
Progestin	7	40	33
Histamine-1 Receptor Antagonist	5	8	3
Factor Xa Inhibitor	39	23	-16
alpha-Adrenergic Blocker	41	15	-26
Nonsteroidal Anti-inflammatory Drug	1	1	0
Opioid Agonist	27	27	0
Biguanide	13	7	-6
Anti-epileptic Agent	18	37	19
Histamine-2 Receptor Antagonist	22	18	-4
Mood Stabilizer	16	35	19
Muscle Relaxant	37	32	-5
Sigma-1 Agonist	28	39	11
Atypical Antipsychotic	21	41	20
HMG-CoA Reductase Inhibitor	23	3	-20
Leukotriene Receptor Antagonist	24	21	-3
Benzodiazepine	15	29	14
Antihistamine	29	38	9
Tricyclic Antidepressant	30	28	-2

epc_class	rank_under 40	rank_over 60	rank_change
Progesterone	38	22	-16

Supplemental Table S12

Rank order of medications in males and females

rank	epc_class	top_med_classes_%_male	epc_class	top_med_classes_%_female
1	Nonsteroidal Anti-inflammatory Drug	29.76	Nonsteroidal Anti-inflammatory Drug	27.83
2	HMG-CoA Reductase Inhibitor	14.37	l-Thyroxine	21.39
3	Platelet Aggregation Inhibitor	14.13	Serotonin Reuptake Inhibitor	13.66
4	Histamine-1 Receptor Antagonist	10.03	Histamine-1 Receptor Antagonist	12.16
5	Serotonin Reuptake Inhibitor	9.23	Central Nervous System Stimulant	7.36
6	Angiotensin 2 Receptor Blocker	8.99	Platelet Aggregation Inhibitor	7.2
7	Central Nervous System Stimulant	8.9	Proton Pump Inhibitor	6.53
8	Biguanide	8.59	Aminoketone	5.92
9	Proton Pump Inhibitor	8	Angiotensin 2 Receptor Blocker	5.41
10	beta-Adrenergic Blocker	7.42	Estrogen	5.3
11	l-Thyroxine	7.08	Biguanide	5.07

rank	epc_class	top_med_classes_%_male	epc_class	top_med_classes_%_female
12	Calcium Channel Blocker	6.65	HMG-CoA Reductase Inhibitor	4.99
13	Dihydropyridine Calcium Channel Blocker	6.37	Corticosteroid	4.96
14	Corticosteroid	4.43	beta-Adrenergic Blocker	4.71
15	Thiazide Diuretic	4.12	Serotonin and Norepinephrine Reuptake Inhibitor	4.3
16	Angiotensin Converting Enzyme Inhibitor	3.94	Progestin	3.68
17	alpha-Adrenergic Blocker	3.51	Thiazide Diuretic	3.38
18	Aminoketone	2.99	Calcium Channel Blocker	3.37
19	Xanthine Oxidase Inhibitor	2.59	Dihydropyridine Calcium Channel Blocker	3.01
20	Serotonin and Norepinephrine Reuptake Inhibitor	2.15	Progesterone	2.98
21	Methylxanthine	2.12	Benzodiazepine	2.46
22	Benzodiazepine	2.06	Methylxanthine	2.33
23	Opioid Agonist	2.06	alpha-Adrenergic Agonist	2.3

rank	epc_class	top_med_classes_%_male	epc_class	top_med_classes_%_female
24	5-alpha Reductase Inhibitor	1.97	Leukotriene Receptor Antagonist	2.2
25	beta2-Adrenergic Agonist	1.82	Histamine-2 Receptor Antagonist	2.11
26	alpha-Adrenergic Agonist	1.72	beta2-Adrenergic Agonist	2.02
27	Histamine-2 Receptor Antagonist	1.6	Opioid Agonist	1.9
28	Factor Xa Inhibitor	1.48	l-Triiodothyronine	1.88
29	Leukotriene Receptor Antagonist	1.39	Mood Stabilizer	1.83
30	Sulfonylurea	1.39	Tricyclic Antidepressant	1.79
31	Androgen	1.32	Anti-epileptic Agent	1.7
32	P2Y12 Platelet Inhibitor	1.32	Aldosterone Antagonist	1.53
33	Peroxisome Proliferator Receptor alpha Agonist	1.23	Angiotensin Converting Enzyme Inhibitor	1.24
34	Mood Stabilizer	1.14	Herpes Simplex Virus Nucleoside Analog DNA Polymerase Inhibitor	1.17

rank	epc_class	top_med_classes_%_male	epc_class	top_med_classes_%_female
35	Sympathomimetic-like Agent	1.14	Antihistamine	1.14
36	Phosphodiesterase 5 Inhibitor	1.11	Muscle Relaxant	1.07
37	Atypical Antipsychotic	1.11	Atypical Antipsychotic	1.07
38	Anti-epileptic Agent	1.08	Serotonin-1b and Serotonin-1d Receptor Agonist	1.04
39	Sigma-1 Agonist	1.02	Sigma-1 Agonist	1.04
40	Antihistamine	0.92	gamma-Aminobutyric Acid A Receptor Positive Modulator	0.88
41	alpha-1 Adrenergic Agonist	0.92	Osmotic Laxative	0.83
42	Insulin Analog	0.92	Tetracycline-class Drug	0.83
43	Dietary Cholesterol Absorption Inhibitor	0.86	Antirheumatic Agent	0.79
44	Dipeptidyl Peptidase 4 Inhibitor	0.86	Expectorant	0.78
45	Muscle Relaxant	0.83	Sympathomimetic Amine Anorectic	0.73

rank	epc_class	top_med_classes_%_male	epc_class	top_med_classes_%_female
46	Sodium-Glucose Cotransporter 2 Inhibitor	0.83	Opioid Antagonist	0.71
47	Thiazide-like Diuretic	0.77	Antimalarial	0.7
48	Expectorant	0.77	Factor Xa Inhibitor	0.66
49	Cholinergic Nicotinic Agonist	0.74	alpha-1 Adrenergic Agonist	0.63
50	gamma-Aminobutyric Acid A Receptor Positive Modulator	0.74	Progestin-containing Intrauterine System	0.61

Supplemental Table S13

Change in rank order of EPC classes between males and females

epc_class	rank_male	rank_female	rank_change
l-Thyroxine	11	2	-9
l-Triiodothyronine	38	28	-10
Proton Pump Inhibitor	9	7	-2
Thiazide Diuretic	15	17	2
Aminoketone	18	8	-10
Central Nervous System Stimulant	7	5	-2
Angiotensin Converting Enzyme Inhibitor	16	33	17
Angiotensin 2 Receptor Blocker	6	9	3
Corticosteroid	14	13	-1
Serotonin and Norepinephrine Reuptake Inhibitor	20	15	-5
Herpes Simplex Virus Nucleoside Analog DNA Polymerase Inhibitor	35	34	-1
beta2-Adrenergic Agonist	24	26	2
Dihydropyridine Calcium Channel Blocker	13	19	6
alpha-Adrenergic Agonist	25	23	-2
Calcium Channel Blocker	12	18	6
Platelet Aggregation Inhibitor	3	6	3
Aldosterone Antagonist	37	32	-5
beta-Adrenergic Blocker	10	14	4
Xanthine Oxidase Inhibitor	19	41	22
Methylxanthine	21	22	1

epc_class	rank_male	rank_female	rank_change
Estrogen	40	10	-30
Serotonin Reuptake Inhibitor	5	3	-2
Histamine-1 Receptor Antagonist	4	4	0
Factor Xa Inhibitor	27	39	12
alpha-Adrenergic Blocker	17	40	23
Nonsteroidal Anti-inflammatory Drug	1	1	0
Opioid Agonist	23	27	4
Biguanide	8	11	3
Anti-epileptic Agent	31	31	0
Histamine-2 Receptor Antagonist	26	25	-1
Mood Stabilizer	29	29	0
Muscle Relaxant	34	37	3
Sigma-1 Agonist	32	38	6
Atypical Antipsychotic	30	36	6
HMG-CoA Reductase Inhibitor	2	12	10
Leukotriene Receptor Antagonist	28	24	-4
Benzodiazepine	22	21	-1
Antihistamine	33	35	2
Tricyclic Antidepressant	36	30	-6
Progesterone	39	20	-19

Supplemental Table S14

Rank order of supplements among males and females

rank	supplement	top_supplements_%_male	supplement	top_supplements_%_female
1	multivitamin	26	magnesium	23.77
2	vitamin d	20.71	vitamin d	23.49
3	magnesium	19.77	multivitamin	21.27
4	fish oil	18.47	probiotics	16.36
5	vitamin d3	14.38	vitamin c	15.29
6	vitamin c	14.01	vitamin d3	13.11
7	omega 3	13.45	omega 3	11.59
8	probiotics	10.84	fish oil	10.64
9	vitamin b12	8.8	vitamin b12	10.51
10	zinc	7.97	calcium	9.8
11	curcumin	7.51	curcumin	7.6
12	melatonin	6.02	zinc	7.45
13	vitamin b complex	5.73	melatonin	6.94
14	creatine	5.69	iron	6.63
15	calcium	5.27	vitamin b complex	6.25
16	coenzyme q10	4.74	collagen	4.57
17	glucosamine	3.57	coenzyme q10	3.96
18	krill oil	3.55	vitamin k2	3.55
19	vitamin k2	3.55	biotin	3.39

rank	supplement	top_supplements_%_male	supplement	top_supplements_%_female
20	bcaas	2.4	vitamin b	3.26
21	vitamin b	2.26	glucosamine	2.62
22	ashwagandha	2.24	vitamin e	2.39
23	resveratrol	2.06	ashwagandha	2.39
24	potassium	2.04	vitamin b9	2.33
25	collagen	1.96	potassium	2.21
26	vitamin e	1.94	krill oil	1.96
27	iron	1.8	cod liver oil	1.84
28	cod liver oil	1.76	digestive enzymes	1.68
29	mushrooms	1.72	fiber	1.65
30	psyllium	1.59	selenium	1.64
31	caffeine	1.43	psyllium	1.59
32	chondroitin	1.43	vitamin k	1.43
33	nmn	1.33	milk thistle	1.33
34	spirulina	1.27	iodine	1.3
35	vitamin k	1.25	vitamin b6	1.29
36	fiber	1.25	spirulina	1.28
37	garlic	1.23	5 htp	1.25
38	vitamin b9	1.17	nac	1.25
39	iodine	1.17	evening primrose oil	1.2
40	l theanine	1.15	minerals	1.17

rank	supplement	top_supplements_%_male	supplement	top_supplements_%_female
41	msm	1.09	resveratrol	1.16
42	niacin	0.99	mushrooms	1.14
43	electrolytes	0.97	chondroitin	1.12
44	ala	0.97	dhea	1.01
45	apple cider vinegar	0.97	apple cider vinegar	0.98
46	5 htp	0.97	berberine	0.95
47	digestive enzymes	0.97	msm	0.94
48	ginkgo biloba	0.95	vitamin a	0.9
49	selenium	0.95	cranberry	0.86
50	mct oil	0.93	garlic	0.85

Supplemental Table S15

Rank order of supplements among different age groups

rank	supplement	top_supplements_%_young	supplement	top_supplements_%_mid	supplement	top_supplements_%_old
1	multivitamin	22.84	magnesium	23.51	magnesium	24.74
2	vitamin d	21.95	multivitamin	22.81	vitamin d	23.78
3	magnesium	20.45	vitamin d	22.8	multivitamin	22.7
4	fish oil	15.39	vitamin c	15.59	vitamin d3	19.88
5	probiotics	14.96	probiotics	14.59	vitamin c	16.22
6	vitamin c	13.54	vitamin d3	13.61	calcium	15.8
7	omega 3	12.91	fish oil	11.9	vitamin b12	13.69
8	vitamin d3	11.58	omega 3	11.8	probiotics	13.03
9	vitamin b12	9.04	vitamin b12	9.85	omega 3	11.41
10	zinc	7.93	calcium	8.95	fish oil	11.35
11	curcumin	5.82	curcumin	8.28	curcumin	10.33
12	melatonin	5.82	zinc	7.44	melatonin	8.35
13	iron	5.61	melatonin	6.89	coenzyme q10	7.75
14	calcium	5.42	vitamin b complex	6.42	zinc	7.39
15	vitamin b complex	5.39	iron	5.18	vitamin b complex	6.97

rank	supplement	top_supplements_%_young	supplement	top_supplements_%_mid	supplement	top_supplements_%_old
16	creatine	3.58	coenzyme q10	4.95	vitamin k2	4.74
17	collagen	3.26	collagen	4.19	glucosamine	4.38
18	vitamin b	2.88	vitamin k2	3.92	biotin	3.78
19	vitamin k2	2.71	glucosamine	3.28	vitamin e	3.72
20	krill oil	2.63	vitamin b	2.93	vitamin b	3.18
21	biotin	2.44	ashwagandha	2.54	psyllium	3.12
22	ashwagandha	2.37	potassium	2.52	collagen	3.12
23	vitamin b9	2.3	biotin	2.45	potassium	3.06
24	coenzyme q10	2.22	krill oil	2.44	cod liver oil	2.58
25	glucosamine	2.05	vitamin e	2.36	iron	2.58
26	bcaas	1.94	cod liver oil	1.84	chondroitin	2.34
27	mushrooms	1.79	psyllium	1.71	fiber	2.28
28	vitamin e	1.65	creatine	1.7	krill oil	2.1
29	cod liver oil	1.55	vitamin b9	1.7	vitamin k	1.98
30	caffeine	1.54	selenium	1.69	vitamin b9	1.92

rank	supplement	top_supplements_%_young	supplement	top_supplements_%_mid	supplement	top_supplements_%_old
31	spirulina	1.45	fiber	1.67	resveratrol	1.86
32	5 htp	1.42	digestive enzymes	1.56	ubiquinol	1.62
33	potassium	1.4	resveratrol	1.55	niacin	1.62
34	digestive enzymes	1.3	vitamin k	1.39	vitamin a	1.5
35	resveratrol	1.19	iodine	1.38	dhea	1.44
36	vitamin b6	1.18	chondroitin	1.33	lutein	1.44
37	vitamin k	1.16	spirulina	1.27	digestive enzymes	1.44
38	selenium	1.11	milk thistle	1.25	ashwagandha	1.32
39	fiber	1.09	nac	1.23	iodine	1.26
40	iodine	1.09	mushrooms	1.16	vitamin b6	1.26
41	milk thistle	1.04	vitamin b6	1.15	ala	1.26
42	l theanine	1.04	minerals	1.14	creatine	1.26
43	psyllium	0.99	msm	1.12	apple cider vinegar	1.2
44	minerals	0.97	dhea	1.09	nac	1.2

rank	supplement	top_supplements_%_young	supplement	top_supplements_%_mid	supplement	top_supplements_%_old
45	garlic	0.96	evening primrose oil	1.07	selenium	1.2
46	apple cider vinegar	0.87	5 htp	1.06	berberine	1.2
47	maca	0.87	berberine	1.05	astaxanthin	1.08
48	glutamine	0.85	apple cider vinegar	1.01	msm	1.08
49	alpha brain	0.85	garlic	0.98	ginkgo biloba	1.02
50	nac	0.85	nmn	0.96	flaxseed oil	1.02

Supplemental Table S16

Change in rank order of supplements between young and old subgroups

supplement	rank_under 40	rank_over 60	rank_change
dhea	50	33	-17
caffeine	30	50	20
vitamin b	18	20	2
vitamin b12	9	7	-2
vitamin b9	23	30	7
vitamin k	37	29	-8
vitamin d	2	2	0
probiotics	5	8	3
coenzyme q10	24	13	-11
vitamin b complex	15	15	0
cod liver oil	29	25	-4
iodine	40	35	-5
vitamin d3	8	4	-4
krill oil	20	28	8
multivitamin	1	3	2
resveratrol	35	31	-4
vitamin c	6	5	-1
apple cider vinegar	45	39	-6
vitamin k2	19	16	-3
vitamin b6	36	37	1

supplement	rank_under 40	rank_over 60	rank_change
biotin	21	18	-3
creatine	16	36	20
mushrooms	27	49	22
collagen	17	21	4
fish oil	4	10	6
curcumin	12	11	-1
msm	47	42	-5
omega 3	7	9	2
minerals	43	44	1
iron	13	24	11
psyllium	42	22	-20
fiber	39	27	-12
milk thistle	41	43	2
vitamin e	28	19	-9
bcaas	26	48	22
spirulina	31	47	16
nac	46	40	-6
glucosamine	25	17	-8
selenium	38	38	0
zinc	10	14	4
chondroitin	48	26	-22
calcium	14	6	-8

supplement	rank_under 40	rank_over 60	rank_change
ashwagandha	22	34	12
garlic	44	45	1
potassium	33	23	-10
berberine	49	41	-8
digestive enzymes	34	32	-2
5 htp	32	46	14
magnesium	3	1	-2
melatonin	11	12	1

Supplemental Table S17

Change in rank order of supplements between males and females

supplement	rank_male	rank_female	rank_change
dhea	49	43	-6
caffeine	31	49	18
vitamin b	21	20	-1
vitamin b12	9	9	0
vitamin b9	38	24	-14
vitamin k	35	32	-3
vitamin d	2	2	0
probiotics	8	4	-4
coenzyme q10	16	17	1
vitamin b complex	13	15	2
cod liver oil	28	27	-1
iodine	37	34	-3
vitamin d3	5	6	1
krill oil	18	26	8
multivitamin	1	3	2
resveratrol	23	40	17
vitamin c	6	5	-1
apple cider vinegar	40	44	4
vitamin k2	19	18	-1
vitamin b6	44	35	-9

supplement	rank_male	rank_female	rank_change
biotin	45	19	-26
creatine	14	48	34
mushrooms	29	41	12
collagen	25	16	-9
fish oil	4	8	4
curcumin	11	11	0
msm	39	46	7
omega 3	7	7	0
minerals	47	39	-8
iron	27	14	-13
psyllium	30	31	1
fiber	34	29	-5
milk thistle	48	33	-15
vitamin e	26	23	-3
bcaas	20	50	30
spirulina	33	36	3
nac	50	37	-13
glucosamine	17	21	4
selenium	43	30	-13
zinc	10	12	2
chondroitin	32	42	10
calcium	15	10	-5

supplement	rank_male	rank_female	rank_change
ashwagandha	22	22	0
garlic	36	47	11
potassium	24	25	1
berberine	46	45	-1
digestive enzymes	42	28	-14
5 htp	41	38	-3
magnesium	3	1	-2
melatonin	12	13	1

Section S1. Supplementary text on Prompt usage.

Prompt Usage

Medication and supplement identification were separately performed using the following prompts in an automated natural-language text processing pipeline built around Gemini 2.5 Flash. Sets of unique participant medication entries (mCC app entries, self-identified by the participant as containing a medication) were given to Gemini as a methodology for transforming a participant's natural language log to a usable set of medication or supplement related phrases. These prompts included instructions on how to perform simple spelling correction and either medication, or supplement phrase recognition, depending on the prompt used. Details of exact typo correction, normalization, and examples can be found within the prompts.

Medication Prompts.

Parse the following list of user-provided medication entries, extracting and normalizing any specific medications described.

Input Medication Entries (Batch):
{input_entries}

Instructions (Apply to EACH entry individually):

1. Identify Concrete Medications
• Extract only **specific** prescription or over-the-counter (OTC) medications.
This includes:
- Generic names (e.g., 'lisinopril', 'metformin', 'nystatin')
- Brand names (e.g., 'zoloft', 'vicodin', 'flonase')

- common clinical abbreviations (e.g., 'ASA' → 'aspirin', 'APAP' → 'acetaminophen, 'HCTZ' -> 'hydrochlorothiazide',
'T3' → 'liothyronine', 'T4' → 'levothyroxine')

• **Exclude** vague or non-specific terms:
- Drug class names: 'antibiotic'
- descriptive or contextual phrases: 'blood pressure meds', 'diabetes medications', 'sleeping pill'
- standalone formulation words: 'cream', 'inhaler', 'patch', 'tablet', 'pill'

• **Exclude** the following categories entirely:
- Foods, beverages, herbs: (e.g., 'coffee', 'green tea', 'turmeric', 'sandwich')
- Nutritional supplements and vitamins (e.g., 'B12', 'vitamin D3', 'fish oil', 'magnesium')
- Recreational or illicit substances (e.g., 'weed', 'CBD')

• If a term is ambiguous, or unclear (e.g., 'methylph'), **omit it entirely** unless you are highly confident of its intended medication name

• Do not hallucinate or guess. If you are not confident in the normalization, omit the phrase.

---

2. Normalize Brand Names (Convert to Generics)

• Normalize **all brand names** - whether single-ingredient or combination - to their corresponding **generic active ingredient(s)**
- Example: 'Tylenol' → 'acetaminophen'
- Example: 'Zyrtec-D' → 'cetirizine/pseudoephedrine'

• If the brand contains **multiple ingredients**, decompose into all known generic components, separated with a '/'
- Example: 'Tylenol PM' → 'acetaminophen/diphenhydramine'
- Example: 'Excedrin' → 'acetaminophen/aspirin/caffeine
- Example: 'Allegra D' → 'fexofenadine/pseudoephedrine
- Example: 'Co-Diovan' → 'valsartan/hydrochlorothiazide
- Example: 'Bactrim' → 'sulfamethoxazole/trimethoprim'

Only decompose if the brand is known to be a combination drug. Avoid guessing or inventing new combinations.

• If the brand is **not available in the U.S.**, but you recognize the active ingredient(s), normalize to the generic form.
- Example: 'Euthyrox' → 'levothyroxine'
- Example: 'Konverge' → 'amlodipine/olmesartan'
- Example: 'Fostair' → 'beclomethasone/formoterol'
- Example: 'Zenaro' → 'levocetirizine'
- Example: 'ipramol' → 'salbutamol/ipratropium'
- Example: 'Alvedon' → 'acetaminophen'
- Example: 'Colazide' → 'balsalazide'
- Example: 'novalgin' → 'metamizole'
- Example: 'apohydro' → 'hydrochlorothiazide'
- Example: 'macmiror' → 'nifuratel'
- Example: 'zoely' → 'nomegestrol/estradiol'
- Example: 'Dafalgan' → 'acetaminophen'
- Example: 'Buscopan' → 'butylscopolamine'
- Example: 'Augmentin' → 'amoxicillin/clavulanate'

- Example: 'Zaditen' → 'ketotifen'
- Example: 'Neobrufen' → 'ibuprofen'
- Example: 'Toplexil' → 'oxomemazine'
- Example: 'Loratadina' → 'loratadine'

• If a **partial brand**, shorthand, international brand, or mispelled brand is confidently recognizable, normalize it.
- Example: 'Advilpm' → 'ibuprofen/diphenhydramine'
- Example: 'candasarten' → 'candesartan'
- Example: 'atorva' → 'atorvastatin'

• If a brand is **ambiguous**, obscure, or unfamiliar - and you are **not confident** in the ingredients - omit or preserve the brand name as is.

• **Omit salt forms, formulation suffixes, and non-therapeutic descriptors**
Normalize medication names to their base ingredient only:
- Example: 'amlodipine besylate' → 'amlodipine'
- Example: 'metoprolol succinate' → 'metoprolol'
- Example: 'phenylephrine HCl → 'phenylephrine'
- Example: 'bupropion XL' → 'bupropion'
- Example: 'thyroid desiccated' -> 'thyroid'
- Example: 'diltiazem XR' → 'diltiazem'
- Example: 'estradiol valerate' → 'estradiol'
- Example: 'valproic acid' → 'valproate'

Exclude suffixes unless they distinguish different ingredients.

---

3. Include OTC Medications with Defined Therapeutic Ingredients

• Include over-the-counter (OTC) medications that have a known active ingredient and are intended for a therapeutic effect
- Example: 'miralax' → 'polyethylene glycol 3350'
- Example: 'tums → 'calcium carbonate'
- Example' 'Melatonin' → 'melatonin'
- Example 'Sudafed' → 'pseudoephedrine'
- Example 'Zyrtec-D' → 'cetirizine/pseudoephedrine'

• OTC items should be included as long as they have a RxNorm compatible ingredient.

• **Do not include** foods, nutritional supplements, vitamins, or general wellness products.

---

4. Handle Typos, Abbreviations, and Shorthand Carefully

• Correct **obvious mispellings** or minor typos before mapping to the generic term.
- Example: 'candesarten' → 'candesartan'
- Example: 'methylpenidate'→ 'methylphenidate'
- Example: 'hydrocholorothiazide' → 'hydrochlorothiazide'
- Example: 'Amour thyroid' → 'thyroid'
- Example: 'Advilpm' → 'ibuprofen/diphenhydramine'

• Correct **partial-name tokens** and shorthand if they confidently map to a known medication.
- Example: 'atorva' → 'atorvastatin'

    - Example: 'thyroxine' → 'levothyroxine'

  • **Discard** ambiguous, incomplete, or shorthand tokens if their intended meaning is unclear or could refer to multiple drugs.
    - Example: discard 'statin', 'methylph', 'blood pressure med', 'diabetes med', etc...

  • **Whitelist and expand** only approved medical abbreviations that are not salt forms or formulation suffixes.
    - Example: 'ASA' → 'aspirin'
    - Example: 'APAP' → 'acetaminophen'
    - Example: 'HCTZ' → 'hydrochlorothiazide
    - Example: 'T4' → 'levothyroxine'

  • If the abbreviation, partial token, or typo **could reasonably map to multiple medications**, **omit it entirely**.
---

5. Handle Parentheticals, Synonyms, and Duplicate Mentions

  • Use contextual clues to determine if a phrase is a duplicate mention or explanatory phrase.
    - Example: 'Thyroid T3' → 'liothyronine' ('thyroid' may be an explanatory phrase describing for what 'T3' refers to)

  • If an entry contains a **paranthetical synonym** or **alternative name** (e.g., 'acetaminophen (Tylenol)'), extract and output **only one canonical term**, prefer the generic name when possible.
    - Example: 'acetaminophen (Tylenol) → 'acetaminophen'
    - Example 'Prilosec OTC (omeprazole) → 'omeprazole'

  • If both the generic and brand name appear separately (e.g., 'acetaminophen tylenol), **output only one canonical term**, prefer the generic.

  • **Do not output duplicates**
    - Each medication name should only appear once in the final list for that entry
    - If the entry has duplicate medications (e.g., 'insulin', 'insulin') omit the duplicate

  • If the synonym is unclear or there is **no clear mapping**, prioritize the **known generic**. If no clear generic can be identified, omit the ambiguous synonym.
---

6. Remove Dosage, Quantity, and Descriptive Noise

• Strip numeric dosages, strength units (mg, mcg, mL, IU, etc.), and administration, route or frequency descriptors.
Do not include any of the following in the output:
  - Dosages: '50 mg', '200 mcg', '1 tablet', '2 pills', '500 IU'
  - Units: 'mg', 'g', 'mL', 'tabs', 'caps', 'pills'
  - Frequency measurements: 'once daily', '2x', 'every night', 'as needed'
  - Route or purpose: 'for pain', 'for sleep', 'taken after lunch'
  - Quantity descriptors 'x2', '3 pills', 'tablet and a half'
  - Contextual descriptions: 'morning meds', 'night dose', 'on weekends'

• The **only valid output** is the **clean, normalized medication name**, no numerical units, instructions, or timing should be included

• If a description contains **only dosage or usage info**, and no recognizable medication(s), return an empty list

---

8. Additional Example Cases
Use the following examples to guide your behavior for real-world user-entered medication logs:

Input:
'Macmiror, Zoely, Armour Thyroid'
Output:
'["nifuratel", "nomegestrol/estradiol", "thyroid"]'

Input:
'Morning Medicine: Insulin 5 units, Metformin 500mg, Blood Pressure Pills'
Output:
'["insulin", "metformin"]'

Input:
'Tylenol PM, Excedrin, Vitamin C'
Output:
'["acetaminophen/diphenhydramine", "acetaminophen/aspirin/caffeine"]'

Input:
'Candesartan 16Mg, Hydrochlorothiazide 12.5Mg, Cozaar'
Output:
'["candesartan", "hydrochlorothiazide", "losartan"]'

Supplement prompts.

Instructions (Apply to EACH entry individually):

1. Identify Concrete Supplements
• Extract only **specific** supplement items or clearly supplement-like products.
  This includes:
  - vitamins/minerals: "vitamin d", "vitamin b", "magnesium", "zinc", "iron"
  - fats/fatty acids: "fish oil", "omega 3", "krill oil"
  - botanicals: "turmeric", "ashwagandha", "ginseng"
  - amino acid / performance aids: "creatine", "beta alanine", "citrulline"
  - probiotics: "probiotics", "lactobacillus"
  - fiber supplements: "psyllium", "metamucil"
  - electrolytes/hydration mixes: "electrolytes", "hydration salts"
  - common named supplements: "glucosamine", "collagen", "chondroitin"

• EXCLUDE vague or non-specific terms like:
  - "my pills", "morning stack", "immune support", "for gut health"
  - general health goals: "sleep", "energy", "focus", "detox"
  - formulation-only words: "pill", "capsule", "tablet", "gummy", "powder", "drops"
  - marketing or wellness phrases: "complex", "formula", "boost", "blend", "active", "health"
  - organ-based terminology that doesn't clearly refer to a supplement

• EXCLUDE the following entirely:
  - prescription/OTC medications or drug-like terms (e.g., "tylenol", "metformin", "ibuprofen")
  - foods/meals/beverages (e.g., "lunch", "taco", "coffee", "water", "juice")
  - meal-like supplements and meal replacement products (e.g., "protein shake", "ensure")
  - lone condition or body-system labels (e.g., "thyroid", "cholesterol", "immune", "energy")

If a candidate is ambiguous (could be food or supplement) and you are not highly confident it is a supplement, OMIT it.
---
2. Normalize Each Extracted Item To Common Names.
• Spell check and correct typos
  - Example: "vitmin D" → "vitamin d"
  - Example: "ginsen" → "ginseng"
  - Example: "l glutamin" → "l glutamine"

• Normalize synonyms and shorthand to a canonical term. Prefer broader terminology for vitamins, but retain any numbered vitamin specificity when appropriate.
Use simplified names for minerals. Retain common abbreviations.:
  - Example: "d3" → "vitamin d3"
  - Example: "b12" → "vitamin b12"
  - Example: "vitamin b" → "vitamin b"
  - Example: "b100" → "vitamin b complex"
  - Example: "omega-3", "omega 3", "n-3" → "omega 3"
  - Example: "fishoil" → "fish oil"
  - Example: "mag" (if clearly magnesium) → "magnesium"
  - Example: "nicotinamide mononucleotide → "nmn"
  - Example: "zinc picolinate" → "zinc"
  - Example: "collagen hydrolysate" → "collagen"
  - Example: "gamma aminobutyric acid" → "gaba"

• Generic supplement categories and phrases (e.g., "probiotic", "fiber", "electrolytes", "vitamin", "supplement") may be output
 only if clearly intended as a supplement and not as a food.

• DO NOT infer ingredients based on common knowledge, marketing associations, or typical formulations.
  - Example: "herbal blend" → []
  - Example: "joint support formula" → []

• If a formula or product name explicitly contains the name(s) of supplement ingredients, those ingredients may be extracted, provided no additional ingredients are inferred.
• If an item is a product or brand name without an explicit ingredient in the text it should be kept as is unless:
  (a) the ingredient(s) are literally present in or very strongly inferred by the product name
  (b) the brand name is strongly and uniquely associated with a single ingredient category or single use
  In these cases, convert to known ingredient. If conversion would require knowing the product's formulation, omit it.
  - Example: "EmergenC" → "vitamin c" (brand name infers "vitamin c" so it can be kept)
  - Example: "OmegaBrite" → "omega 3"
  - Example: "Omega3D" → "omega 3", "vitamin d"
  - Example: "Garden of Life" → [] (brand names should be excluded if they cannot be mapped to a single supplement
  - Example: "ag1" → "ag1"
  - Example: "NatureMade D3" → "vitamin d3"
  - Example: "Metamucil" → "psyllium"

• Avoid over-normalization of important qualifiers.
  - Example: "magnesium glycinate" → "magnesium" (complex forms of vitamins, minerals, etc. should be shortened)
  - Example: "vitamin d" and "vitamin k" should not be normalized together as they are different vitamins

• Do not combine supplements into combinations unless:
  (a) AND the combination is written by the user without separators (e.g. "ABC")
  (b) OR written with a "/" (e.g. "A/B/C")
  - Example: "vitamin d and k2" → "vitamin d, vitamin k2"
  - Example: "vitamin b complex" should not be expanded into individual vitamins
  - Example: "vitamins a d k" → "vitamin a", "vitamin d", "vitamin k"

• Individual vitamins and minerals should always be output as separate items. DO not combine vitamin tokens unless explicitly
written as a combined string.

• Vitamin complexes should remain as their own single item (e.g. "vitamin b complex")
  - Example: "vitamin b complex plus c" → "vitamin b complex, vitamin c"

• Use context to infer what adjectives modify. Phrases like 'multi' may be abbreviations for 'multivitamin' on their own
but may instead modify the nearest noun when paired with other phrases.
  - Example: "multi" → "multivitamin" (may plausibly refer to multivitamin on its on)
  - Example: "mutli probiotic" → "probiotic" (multi is more likely to be a modifier to the phrase, and not its own mention)

---

3. Handle Duplicates and Parentheticals
• If the same supplement is mentioned multiple times in one entry, output it once.
• If a vitamin appears as a complex and as a lone vitamin in the same entry output each once if the lone vitamin is properly specified
  - Example: "vitamin b complex, vitamin b6" → "vitamin b complex, "vitamin b6"

• If a synonym appears in parentheses, output only one canonical term:
  - Example: "vitamin d (d3)" → "vitamin d3"

---

4. Boundary / Edge Cases
• Protein powders, protein shakes, meal replacement shakes should be excluded, while creatine should be included
• Drinks such as 'gatorade' and caloric hydration powders should be excluded. If electrolytes are listed generically (e.g., "electrolytes"), include only if non-caloric and not a beverage.
• If individual minerals are listed (e.g., sodium, potassium, magnesium), list them individually and do not combine.

---

5. Remove Dosage, Quantity, and Descriptive Noise

• Strip numeric dosages, strength units (mg, mcg, mL, IU, etc.), and administration, route or frequency descriptors.
Do not include any of the following in the output:
  - Dosages: '50 mg', '200 mcg', '1 tablet', '2 pills', '500 IU'
  - Units: 'mg', 'g', 'mL', 'tabs', 'caps', 'pills'
  - Frequency measurements: 'once daily', '2x', 'every night', 'as needed'
  - Route or purpose: 'for pain', 'for sleep', 'taken after lunch'
  - Quantity descriptors 'x2', '3 pills', 'tablet and a half'
  - Contextual descriptions: 'morning supplements', 'night dose', 'on weekends', "formula", "formulation", "combo"

• The **only valid output** is the **clean, normalized supplement name**, no numerical units, instructions, or timing should be included
• If a description contains **only dosage or usage info**, and no recognizable supplement(s), return an empty list
• For each supplement candidate if multiple interpretations are plausible, choose the more conservative one that produces fewer supplements.

---

6. Additional Example Cases
Use the following examples to guide your behavior for real-world user-entered logs. When in doubt, omit rather than guess.
Do not infer ingredients beyond what is explicitly or unambiguously encoded:

Input:
'Iron Supplement, vitamin C, ginger Lemon', "b100", "osteobio"
Output:
"iron", "vitamin c", "vitamin b complex"

Input:
'glucosamine and Msm, vitamin b12', "emergenc", "athletic greens"
Output:
"glucosamine", "msm", "vitamin b12", "vitamin c", "ag1"

Input:
'Microgestin Fe, 100mg Sertraline, 2 Capsules Of Adrecor, 150mg Wellbutrin, 10mg Allertec'
Output:
"adrecor"

Input:
'Vitamin D3, Ginkgo, Coenzyme Q10, Moringa Brain Greens, Super10 Pro, Glucosamine, Omega 3, Optitumeri'
Output:
"vitamin d3", "ginkgo biloba", "coenzyme q10", "moringa", "glucosamine", "omega 3", "turmeric"
